# Insights into the translational activation mechanisms of the *COX1* mRNA in yeast mitochondria

**DOI:** 10.1242/jcs.263694

**Published:** 2025-09-02

**Authors:** Angelica Zamudio-Ochoa, Yolanda Camacho-Villasana, Ulrik Pedroza-Dávila, Aldo E. García-Guerrero, Xochitl Pérez-Martínez

**Affiliations:** ^1^ Napigen Inc, Wilmington, DE 19803, USA; ^2^ Departamento de Genetica Molecular, Instituto de Fisiologia Celular, Universidad Nacional Autonoma de Mexico, CDMX, 04510, Mexico; ^3^ Department of Biochemistry, University of Utah School of Medicine, Salt Lake City, UT 84112, USA

**Keywords:** *COX1* mRNA, Mitochondria, Mitoribosome, Mss51, Pet309, Translation

## Abstract

Mitochondrial translation is a crucial regulatory step in mitochondrial genome expression. In *Saccharomyces cerevisiae*, translational activators are believed to bind to the 5′ UTRs of their target mRNAs to position the mitochondrial ribosome at the start codon. Pet309 and Mss51 are translational activators of *COX1* mRNA, which encodes subunit one of cytochrome *c* oxidase. Pet309 physically interacts with *COX1* mRNA, but no direct interaction of Mss51 with its target mRNA has been detected. Currently, the mechanisms underlying translational activation of *COX1*, or any other mitochondrial gene, remain poorly understood. To explore in depth the mechanism of *COX1* mRNA translational activation, we studied the association of Pet309 and Mss51 with the mitochondrial ribosome. Both Pet309 and Mss51 interact with the mitoribosome regardless of the presence of *COX1* mRNA or of each other. The association of Pet309 with the ribosome and with *COX1* mRNA depends on its N-terminal domain. These findings indicate that Pet309 and Mss51 stably interact with the mitoribosome independently of active translation. By integrating our data with previously published research, we propose a new mechanism of *COX1* mRNA translation activation.

## Teaser

**Highlighted Article:** Yeast mitochondrial mRNAs require translational activators by an almost unknown mechanism. Based on our findings on Mss51 and Pet309 function, we present a new model for translation of the *COX1* mRNA.

## INTRODUCTION

Since the discovery of the mitochondrial genome in the 1960s (Nass and Nass, 1963; Schatz et al., 1964), extensive research has been conducted to unravel the molecular mechanisms underlying its maintenance, expression and regulation. Among these mechanisms, translation stands out as a crucial regulatory step in mitochondrial genome expression (Couvillion et al., 2016; Fontanesi, 2013; Richter-Dennerlein et al., 2016). Despite significant progress, our understanding of this process remains incomplete. In *Saccharomyces cerevisiae*, the mitochondrial genome encodes two ribosomal RNAs, 22 transfer RNAs, the 9S RNA (a component of RNase P) and eight proteins (Foury et al., 1998). Seven of these mitochondria-encoded proteins are integral subunits of the respiratory complexes and ATP synthase, and their translation is tightly regulated and synchronized with the assembly of the nuclear-encoded subunits (Couvillion et al., 2016). This phenomenon does not seem to exist in mammalian mitochondria (Soto et al., 2022).

Yeast mitochondrial mRNAs feature long 5′ untranslated regions (UTRs), essential for translation regulation via translational activators, although the precise regulation mechanism remains unclear. It is hypothesized that these translational activators help position mitochondrial ribosomes on the start codon of the mRNA (Fox, 1996). Each mitochondrial mRNA has a unique set of translational activators (Herrmann et al., 2013). Furthermore, the majority of yeast mitochondrial mRNAs depend on at least one translational activator containing pentatricopeptide repeat (PPR) motifs, which function as RNA-binding domains (Lipinski et al., 2011). PPR proteins belong to the alpha-solenoid structure tandem repeat proteins group (STRPs) (Arrias et al., 2024). PPR proteins consists of multiple 35-amino-acid degenerate repeats, with each repeat forming two antiparallel α-helices (Ringel et al., 2011). Each repeat binds one nucleotide of its target mRNA for specific RNA recognition (Filipovska and Rackham, 2013). Currently, 15 PPR proteins have been identified in yeast, all of which are localized within mitochondria and are essential in RNA metabolism functions, including transcription, stability and translation of mRNAs (Herbert et al., 2013; Lipinski et al., 2011).

The PPR protein Pet309 is a translational activator of the *COX1* mRNA (Manthey and McEwen, 1995). This mRNA encodes Cox1, the largest and core subunit of cytochrome *c* oxidase (C*c*O) (Scott, 1995). C*c*O is indispensable for respiration, serving as the terminal electron acceptor in the electron transport chain and catalyzing the reduction of molecular oxygen to water. Pet309 specifically acts on the 5′UTR of the *COX1* mRNA to activate its translation. We previously reported the cooperative action of the PPR motifs of Pet309 on *COX1* mRNA binding (Zamudio-Ochoa et al., 2014). Furthermore, Pet309, along with other translational activators, has been identified in mitochondrial ribosome co-purifications (Kehrein et al., 2015).

In addition to Pet309, Mss51 is also a *COX1* mRNA translational activator, despite lacking a detectable physical interaction with the transcript (Zamudio-Ochoa et al., 2014). Mss51 coordinates the synthesis of Cox1 with its assembly into the C*c*O complex (Perez-Martinez et al., 2003). Mss51 physically interacts with the newly synthesized Cox1 and acts as a chaperone, facilitating its assembly into C*c*O. The interaction between Cox1 and Mss51 leads to the formation of translational repressor complexes known as COA complexes (Fontanesi et al., 2011; Mick et al., 2010; Pierrel et al., 2007). These complexes serve as checkpoints during C*c*O assembly, halting the translational activator function of Mss51. When C*c*O assembly is completed, Mss51 is released from the COA complexes, initiating a new round of *COX1* mRNA translation. However, if defects in C*c*O assembly occur, Mss51 remains sequestered in the COA complexes, preventing *COX1* mRNA translation (for a review, see Franco et al., 2020).

Despite extensive efforts to elucidate the roles of Pet309 and Mss51 as translational activators, the mechanisms underlying their activity in *COX1* mRNA translation remain elusive. Furthermore, the interplay between these two activators in regulating Cox1 synthesis is poorly understood. Contrary to the proven association of Pet309 with the mitoribosome, studies on the association of Mss51 are inconsistent – some studies found Mss51 associated with high molecular mass complexes (Fontanesi et al., 2010; Verma et al., 2021), whereas another study did not detect it in purified mitoribosomes (Kehrein et al., 2015). To advance our understanding of *COX1* mRNA translation, we investigated the interactions of Pet309 and Mss51 with the mitoribosome. Using sucrose gradient ultracentrifugation, we found that Pet309 and Mss51 independently bind the mitochondrial ribosome regardless of *COX1* mRNA active translation or each other's presence. In addition, we identified the N-terminal region of Pet309 as being crucial for ribosome and *COX1* mRNA binding. Based on these findings and previous data from other groups, we propose a new model for *COX1* mRNA translation.

## RESULTS

### Pet309 constitutively interacts with the mitoribosome independently of the *COX1* mRNA and Mss51

**Fig. 1.**
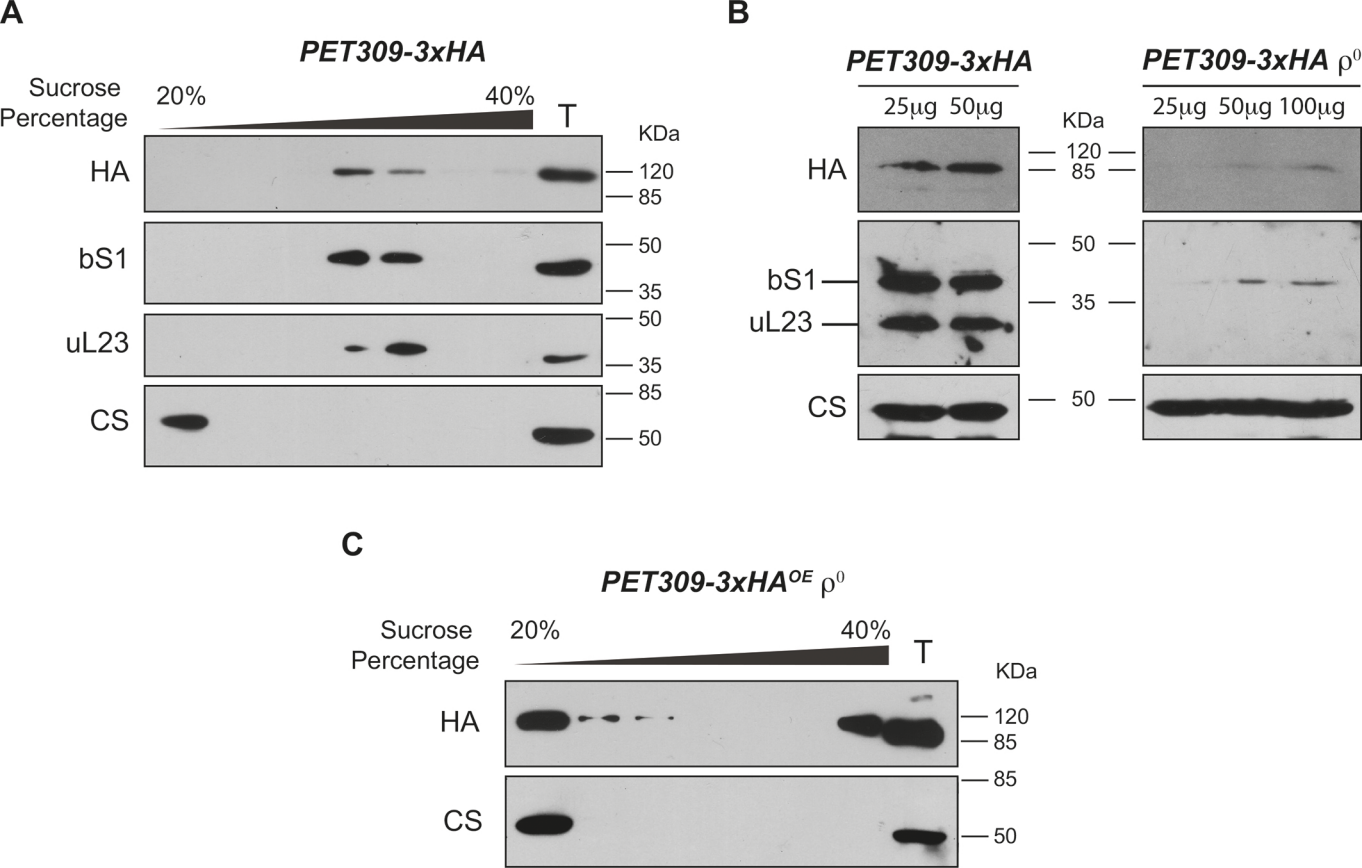
**Pet309 associates with the mitoribosome.** (A) 500 µg of mitochondrial protein from the *PET309-3xHA* strain were lysed with 1% digitonin. Clarified lysate was centrifuged in a discontinuous 20–40% sucrose gradient. Seven fractions were collected and analyzed by SDS-PAGE and western blotting using the indicated antibodies. (B) Western blot analysis with indicated amounts of mitochondrial protein from the *PET309-3xHA* and *PET309-3xHA ρ^0^* strains. (C) Same experiment as shown in A with lysed mitochondria from the *ρ^0^* strain. For efficient immunodetection, a strain overexpressing *PET309-3xHA^OE^* was used in this experiment. In all experiments citrate synthase (CS) was used as loading control or a protein independent form the respiratory chain function. bS1, small mitoribosomal subunit (Mrp51); uL23 large mitoribosomal subunit (Mrp21); T, total fraction equivalent to 7% from the loaded clarified lysate. For all experiments, we used *n*=3 biological replicates. Uncropped blots are shown in Fig. S5.

Translational activators like Pet309 are proposed to position the mitoribosome on the mRNA start codon of its target mRNA (Fox, 1996). Indeed, Pet309 has shown genetical and physical interaction with the *COX1* mRNA (Manthey and McEwen, 1995; Zamudio-Ochoa et al., 2014). In addition, Pet309 was previously detected in a mass spectrometry analysis from isolated mitoribosomes, revealing that Pet309 interacts with the mitochondrial ribosome, and is part of large assemblies termed mitochondrial organization of gene expression (MIOREX) complexes (Kehrein et al., 2015). We looked closer into this interaction and investigated whether the Pet309–mitoribosome association depended on the presence of the *COX1* mRNA and Mss51. To analyze the Pet309–mitoribosome interaction, Pet309 was tagged with three hemagglutinin epitopes at its C-terminal domain (*PET309-3xHA*). This construct was cloned into a centromeric plasmid (CEN). Yeast *pet309Δ* cells were transformed with this plasmid for expression. No respiratory defect in the strain bearing the *PET309-3xHA* construct was observed, as previously reported (Figs S1B and S2A; Tavares-Carreón et al., 2008; Zamudio-Ochoa et al., 2014). To evaluate Pet309 association with the mitoribosome, mitochondria isolated from the *PET309-3xHA* strain were lysed using 1% digitonin, and the clarified lysate was loaded onto a discontinuous sucrose gradient (García-Guerrero et al., 2018). The conditions used in all our experiments preserved the assembled mitoribosome. No EDTA was added to dissociate the subunits (Aufschnaiter et al., 2023). After ultracentrifugation, the gradient was divided into seven fractions and analyzed by western blotting. Given that the integrity of the ribosomes was preserved, we decided to analyze seven fractions from the sucrose gradients instead of the 12 to 15 fractions that are commonly used, which are especially useful when analyzing disassembled subunits (Anderson et al., 2022; Aufschnaiter et al., 2023; Choi and Barrientos, 2021; Hillman and Henry, 2019; Mays et al., 2019). The ribosome migration patterns were similar after analyzing both seven and 14 fractions (Fig. S3). Pet309–3xHA was detected in the same fractions as bS1 (also known as Mrp51) and uL23 (also known as Mrp20), mitoribosomal proteins of the small and large subunits, respectively (Fig. 1A). As expected, citrate synthase (CS), an enzyme from the citric acid cycle unrelated to the respiratory chain, was found in the surface portion of the gradient. Notably, Pet309–3xHA was not detected in top fractions, suggesting that all Pet309 present in mitochondria interacts with the mitoribosome. Interestingly, Pet309–3xHA was almost undetectable by western blotting in a strain lacking mitochondrial DNA (*ρ°*). Given that the large and small rRNAs, as well as the ribosomal protein Var1 are encoded in this genome, a *ρ°* strain has no mitoribosomes. This result suggests that stability of Pet309 depends on the presence of mitoribosomes (Fig. 1B). To have more insights on Pet309 and its interaction with the mitoribosome, we aimed to analyze Pet309–3xHA migration in a *ρ°* strain. Owing to the observed instability of Pet309 in a *ρ°* strain, we cloned *PET309-3xHA* into a 2 μ plasmid for overexpression (*PET309-3xHA^OE^*). In contrast to *CEN* plasmid expression, Pet309–3xHA^OE^ was detected on the surface fraction of the sucrose gradient (Fig. 1C). This finding indicates that Pet309 migration into high molecular mass fractions depends on the presence of the mitoribosome.

**Fig. 2.**
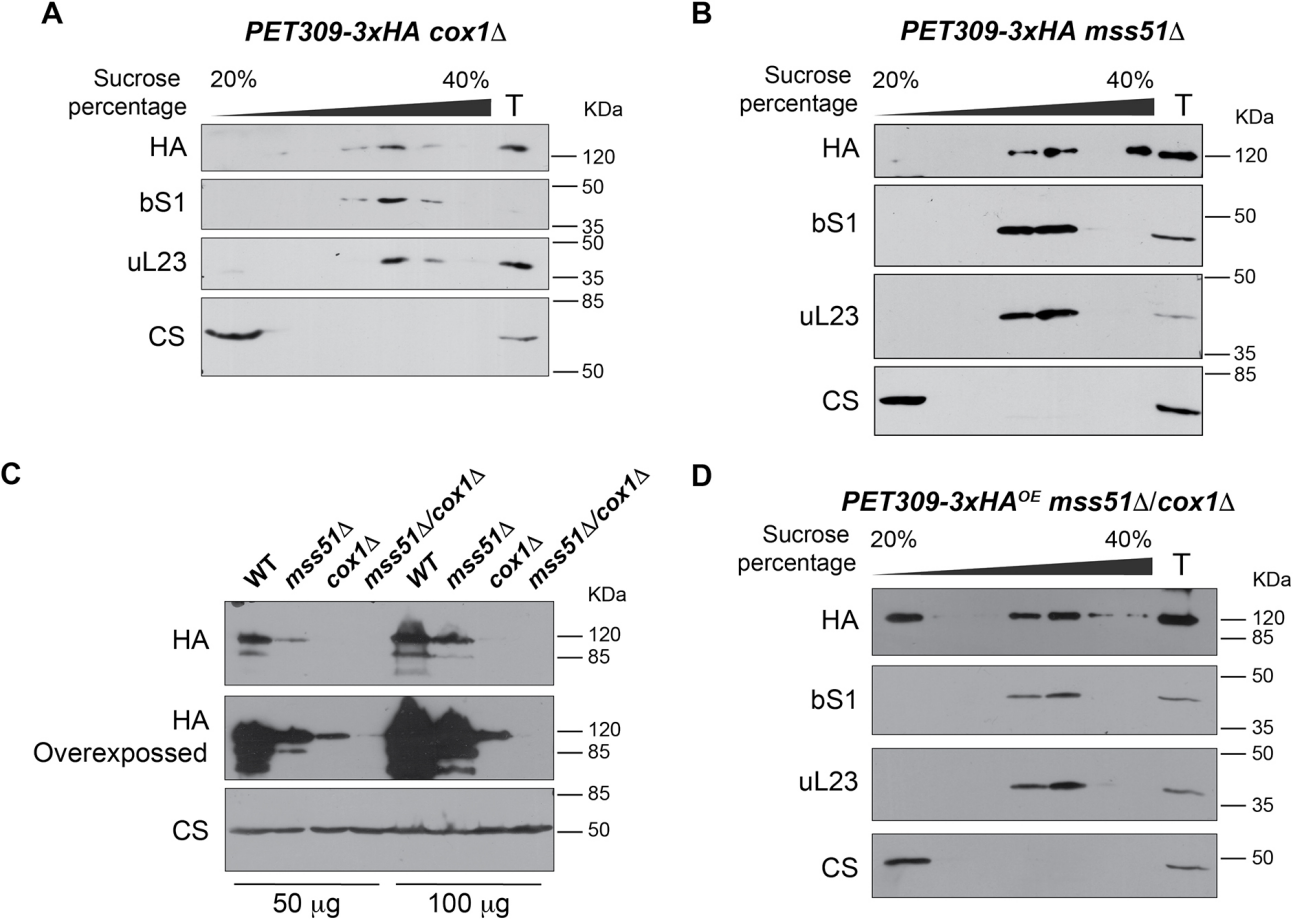
**Pet309 constitutively associates with the mitoribosome.** Mitochondria bearing Pet309–3xHA with the mutants (A) *cox1Δ* or *mss51Δ* were lysed and separated through a sucrose gradient as in Fig. 1. Fractions from the gradient were analyzed by western blotting. (C) Steady state levels of Pet309–3xHA in the indicated mutants were analyzed by western blotting. (D) Western blot analysis from a sucrose gradient separation of the mitochondrial lysate from Pet309–3xHA mitochondria bearing the double mutants *cox1Δ*, *mss51Δ*. Citrate synthase (CS) was used as loading control or a protein independent from the respiratory chain function. bS1, small subunit protein; uL23, large subunit protein; T, Total fraction, equivalent to 7% of the load. For all experiments, we used *n*=3 biological replicates. Uncropped blots are shown in Fig. S5.

Pet309 physically interacts with the *COX1* mRNA (Zamudio-Ochoa et al., 2014), thus Pet309 interaction with the mitoribosome could be mediated by an active translation of its specific mRNA, as previously observed for the *COB* translational activator Cbs2 (Krause-Buchholz et al., 2004). To determine whether Pet309 association with the mitoribosome relies upon the presence of the *COX1* mRNA, we expressed Pet309–3xHA in a strain that lacks the entire *COX1* open reading frame (ORF), including its UTRs (*cox1*Δ). In this strain, the *COX1* gene was deleted from 787 base pairs (bp) upstream of the AUG start codon to 525 bp downstream of the stop codon. We observed that Pet309–3xHA co-migrated with the mitoribosome in *cox1Δ* mitochondria, indicating that the *COX1* mRNA is not required for Pet309 association with the mitoribosome (Fig. 2A).

In contrast to Pet309, Mss51 has not been demonstrated to interact with the *COX1* mRNA, and the mechanisms of how Mss51 activates Cox1 synthesis remains unclear (Zambrano et al., 2007; Zamudio-Ochoa et al., 2014). We hypothesized that Mss51 enable Pet309 association with the mitoribosome, explaining the requirement of Mss51 for *COX1* mRNA translation. To test this hypothesis, we expressed Pet309–3xHA in a *mss51Δ* strain, and analyzed its migration in sucrose gradients. Pet309–3xHA still co-migrated with the mitoribosome in the absence of Mss51, demonstrating that Mss51 is not required for Pet309 association with the mitoribosome (Fig. 2B). Although, steady state levels of Pet309 were already low in *mss51*Δ or *cox1*Δ strains, in the double *mss51*Δ/*cox1*Δ mutant levels of Pet309 were nearly undetectable (Fig. 2C). One explanation could be that Pet309 loses its stability in the absence of Mss51 and the *COX1* mRNA due to its failure to associate with the mitoribosome, as observed in the *ρ°* strain (Fig. 1C). Therefore, we evaluated the association of Pet309 with the mitoribosome in the double mutant *mss51Δ*/*cox1Δ*, but this time by using the *PET309-3xHA^OE^* strain to allow for Pet309 detection. Surprisingly, Pet309–3xHA^OE^ still co-migrated with the mitochondrial ribosome, indicating that neither Mss51 nor the *COX1* mRNA are necessary for Pet309 association with the mitoribosome (Fig. 2D). We observed that some Pet309–3xHA^OE^ was also present in the surface fraction, likely due to the overproduction of Pet309–3xHA and saturation of the mitoribosomes.

In conclusion, our data indicate that Pet309 has a stable and constitutive interaction with the mitoribosome, independent of the presence of Mss51 and the *COX1* mRNA. However, the steady state levels of Pet309 are compromised in the absence of either Mss51, the *COX1* mRNA or the mitoribosomes.

### The N-terminus region of Pet309 is necessary for its interaction with the mitoribosome and with the *COX1* mRNA

**Fig. 3.**
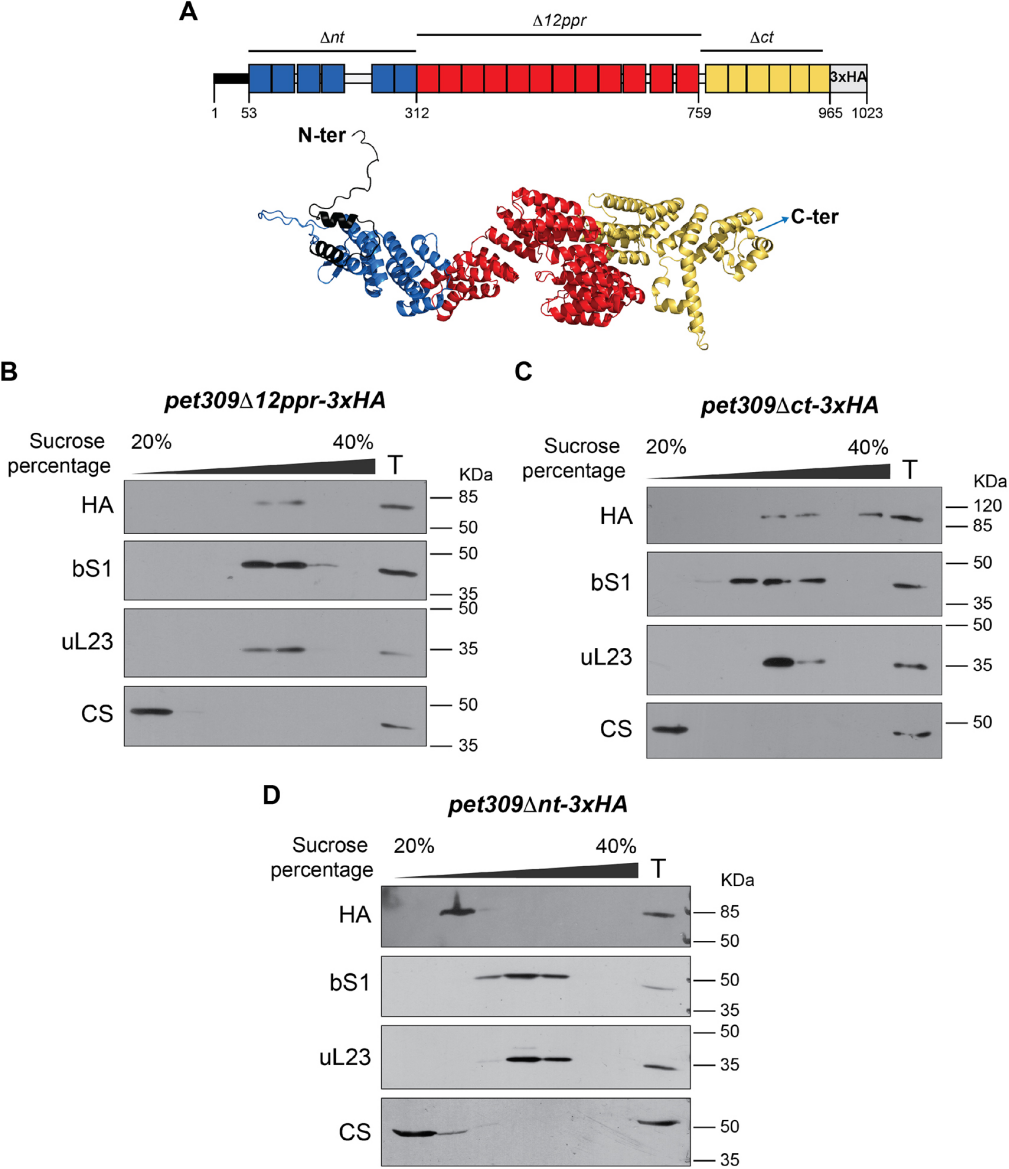
**The N-terminal PPR module of Pet309 mediates interaction with the mitoribosome.** (A) Diagram indicating the three regions form Pet309 that were analyzed in this study. Pet309Δnt lacks residues A53 to Q311, Pet309Δ12ppr lacks residues N312 to N759 and Pet309Δct lacks residues L760 to V962. The triple hemagglutinin epitope (3xHA) was used to immunodetect Pet309 proteins with antibodies as indicated. Bottom panel, a model of Pet309 was downloaded from the AlphaFold database of structural modeling (Jumper et al., 2021). The three proposed modules that conform Pet309 are indicated by colors: N-terminus module, blue; central module bearing 12 PPRs, red; C-terminus module, yellow. The figure was created using Pymol. (B–D) Western blot analysis of the sucrose gradient separation from mitochondria carrying the *pet309Δ12ppr-3xHA* (B), *pet309Δnt-3xHA* (C) and *pet309Δnt-3xHA* (D) constructs. bS1, small subunit protein. uL23, large subunit protein. CS. Citrate synthase. T, Total fraction, equivalent to 7% of the load. For all experiments, we used *n*=3 biological replicates. Uncropped blots are shown in Fig. S6.

As previously indicated, Pet309 is a PPR protein that belongs to the alpha-solenoid structured tandem repeat group of proteins (Arrias et al., 2024). Analysis of the Pet309 sequence by AlphaFold predicted the presence of at least 24 PPR motifs distributed along the entire sequence (Fig. 3A) (Jumper et al., 2021). Three different modules are distinguished and seem to be separated by discrete turns. The previously characterized 12 central PPRs motifs formed one of the three modules (presented in red in Fig. 3A; Zamudio-Ochoa et al., 2014). This region of Pet309 made a clear superhelical structure containing well-ordered PPR motifs, and the structure of this module was predicted by AlphaFold with high confidence score values (Fig. S1A) (Jumper et al., 2021). The N-terminal region (containing six PPR motifs) formed the first module (indicated in blue in Fig. 3A), where the PPR repeats were also well ordered, but created a flatter structure. The last five to six PPR motifs, comprising the Pet309 C-terminus, defined the third module (indicated in yellow in Fig. 3A). This module seems to be less ordered as compared to the central and N-terminus modules, with longer and shorter α-helixes, and in some cases with breaks on some of them.

We previously demonstrated that the lack of the 12 central PPR motifs reduced Pet309 affinity for the *COX1* mRNA, abolishing translation (Zamudio-Ochoa et al., 2014). Therefore, we aimed to evaluate whether Pet309 association with the mitoribosome is similarly affected by the absence of the 12 central PPR motifs. We cloned a *pet309Δ12ppr-3xHA* construct on a centromeric plasmid and transformed into *pet309Δ* cells (Zamudio-Ochoa et al., 2014). This version of the protein lacked the central module from N312 to N759, and affected the growth of the strain on respiratory medium (Zamudio-Ochoa et al., 2014). After centrifuging mitochondrial lysates on a sucrose gradient, Pet309Δ12ppr–3xHA was detected co-migrating with the mitochondrial ribosome (Fig. 3B), indicating that, although the 12 central PPR motifs are essential for *COX1* mRNA translation, they do not play a role in Pet309 association with the mitoribosome.

Pet122 and Cbs2, translational activators of the *COX3* and *COB* mRNAs, respectively, interact with the mitoribosome through their C-terminal regions (Haffter et al., 1990; Krause-Buchholz et al., 2004; McMullin et al., 1990). To determine whether Pet309 C-terminal domain is similarly necessary for its interaction with the mitoribosome, we generated a mutant lacking the last PPR motifs (*pet309*Δ*ct-3xHA*), just downstream of the central PPR domain, from L760 to V962. This region is part of the third PPR module of the Pet309 predicted structure (yellow region in Fig. 3A). Although the association of Pet309Δct–3xHA with the mitochondrial inner membrane was unaltered, it failed to activate *COX1* mRNA translation and negatively impacted the respiratory capacity of the strain (Fig. S1B). Pet309Δct–3xHA co-migrated with uL23 and bS1 in sucrose gradients, indicating that the C-terminal domain does not mediate Pet309 interaction with the mitoribosome (Fig. 3C).

Because the central and C-terminal Pet309 modules were not required for ribosome association, we aimed to evaluate whether the N-terminal region of Pet309 had a role on mitoribosome binding by creating a mutant without the first six PPR domains (*pet309*Δ*nt-3xHA*), from A53 to Q311. This module is predicted to have a more open conformation than the central module (blue module on Fig. 3A). Like its wild-type counterpart, Pet309Δnt-3xHA remained associated with the mitochondrial inner membrane as a peripheral protein but was completely unable to activate Cox1 synthesis, and it failed to keep the respiratory phenotype of the strain (Fig. S2). Remarkably, Pet309Δnt–3xHA did not co-migrate with uL23 and bS1 in the sucrose gradient, and Pet309Δnt-3xHA was detected in surface fractions (Fig. 3D). This result suggests that the ability of Pet309 to bind the mitoribosome resides within its first six predicted PPR domains.

**Fig. 4.**
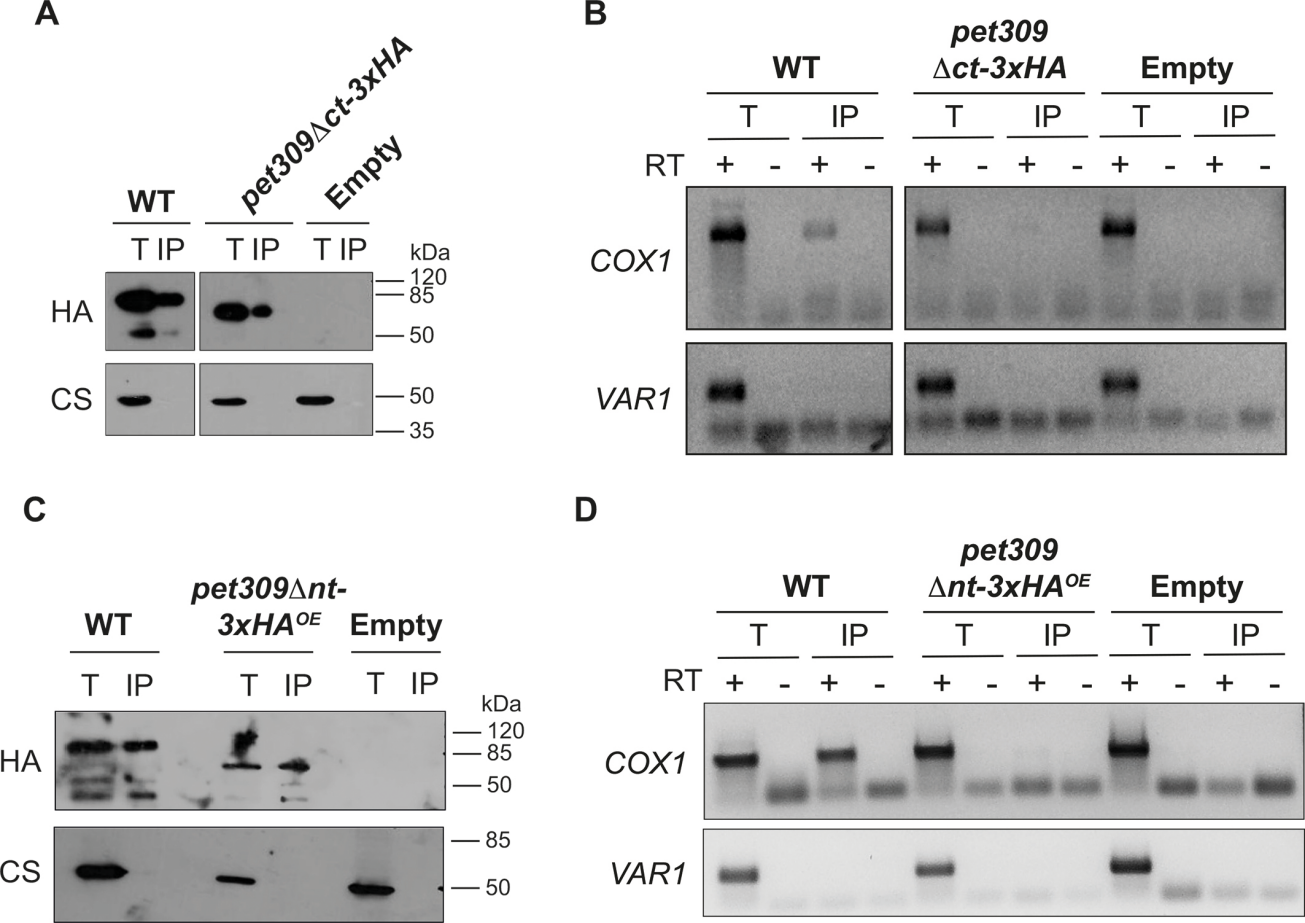
**The N-terminal module of Pet309 is necessary for interaction with the *COX1* mRNA.** Mitochondria from *PET309-3xHA* (WT lanes), (A) *pet309Δct-3xHA* or (C) *pet309Δnt-3xHA^OE^* and the untagged strain were lysed with digitonin. The lysates were subjected to immunoprecipitation with anti-HA antibodies coupled to agarose beads. 5% of the lysate and 25% of the immunoprecipitated (IP) were separated by SDS-PAGE and analyzed by western blotting using an anti-HA antibody (HA) to show the efficiency of immunoprecipitation. An anti-citrate synthase antibody (CS) was used as a negative control. Next, RNA was extracted from the total (T), immunoprecipitated (IP) and supernatant (S) fractions from (B) *pet309Δct-3xHA* or (D) *pet309Δnt-3xHA^OE^* samples. After DNase treatment, cDNA was prepared using reverse transcriptase (RT) (+) and adding primers for *COX1* and *VAR1* genes (Table S2). Samples without RT (−) were included as a control for DNA contamination. Resulting cDNAs were used as a template for PCR amplification of *COX1* and *VAR1* genes and run in an agarose gel. For all experiments, we used *n*=3 biological replicates Uncropped blots are shown in Fig. S6.

As mentioned above, deletion of the 12 central PPR motifs compromised the Pet309–*COX1* mRNA binding. Therefore, we were interested in studying how deletion of the N-terminal module and the C-terminal module of Pet309 affected binding to the *COX1* mRNA. Thus, we performed an RNA-immunoprecipitation (RNA-IP) assay (Zamudio-Ochoa et al., 2014). Mitochondria bearing the *pet309Δct-3xHA* or the *pet309Δnt-3xHA* mutations were solubilized with dodecyl-maltoside. Next, the Pet309 variants were immunoprecipitated with a commercial anti-HA antibody (Fig. 4A). After extensive washing, total RNA was purified from the immunoprecipitates and cDNA was obtained by reverse transcription with specific primers for *COX1* and *VAR1*. The *VAR1* gene was used as a negative control, as Pet309 does not interact with *VAR1* mRNA. The cDNA was further analyzed by PCR to amplify the *COX1* 5′ UTR or *VAR1* 5′ UTR regions. The *COX1* 5′ UTR was successfully amplified in IP fractions from both Pet309–3xHA and Pet309Δct–3xHA. As expected, the *COX1* 5′ UTR was not detected in the untagged Pet309 IP control. Additionally, *VAR1*, was not amplified in any IP fractions (Fig. 4B). In contrast, interaction of Pet309Δnt-3xHA^OE^ with the *COX1* mRNA 5′ UTR was undetectable, suggesting that the N-terminal module of Pet309 is necessary for *COX1* mRNA binding (Fig. 4C,D).

**Fig. 5.**
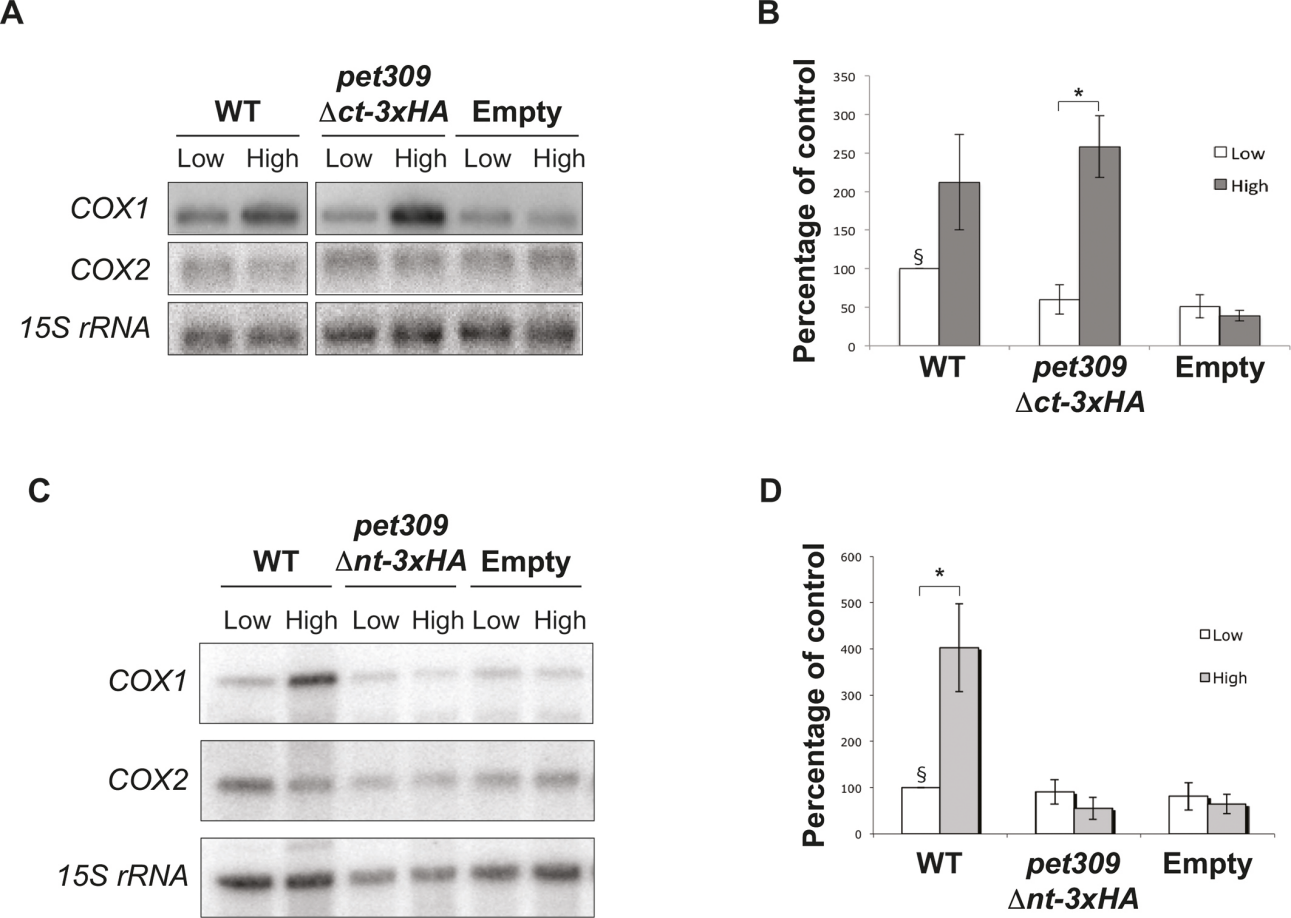
**The N-terminal module from Pet309 is involved in stabilization of the *COX1* mRNA.** The *PET309-3xHA* (WT lanes), *pet309Δct-3xHA* (A) *pet309Δnt-3xHA* (B) were cloned on centromeric plasmid for low copy number (Low) and 2 µ plasmid for high copy number (High) and transformed into a *pet309Δ* strain. An empty plasmid was also transformed and used as negative control. 10 µg of total RNA was analyzed by northern blotting. As a negative control, total RNA from *pet309Δ* strains bearing empty plasmids was also analyzed. The membrane was hybridized with ^32^P-labeled probes complementary to *COX1*, *COX2* and *15S rRNA* probes. (B,D) Quantification of the *COX1* signals from three independent experiments are represented in a bar graph (mean±s.d.). *COX1* signals were normalized against *15S* rRNA and the result from *PET309-3xHA* in low copy plasmid was taken as 100% (§). For all experiments we used *n*=3 biological replicates. *P<0.05 (two-way ANOVA with Bonferroni post-hoc test).

It is well established that Pet309 stabilizes the *COX1* mRNA, and its overexpression leads to an accumulation of *COX1* mRNA, likely due to its capacity to protect the RNA from endogenous nucleases (Fig. S4) (Zamudio-Ochoa et al., 2014). To determine whether the overexpression of Pet309Δct–3xHA or Pet309Δnt–3xHA leads to *COX1* mRNA accumulation, we transformed a *pet309*Δ strain with constructs expressing *pet309*Δ*ct-3xHA* (or *pet309*Δ*nt-3xHA*), *PET309-3xHA* (as a positive control) or an empty vector (as a negative control), from high- or low-copy expression vectors. We purified total RNA from all strains and the accumulation of the *COX1* mRNA was analyzed by northern blotting using ^32^P-labeled oligonucleotides specific for *COX1*, *COX2* (as negative control) and *15S* rRNA (as loading control). Overexpression of Pet309–3xHA and Pet309Δct–3xHA led to an accumulation of *COX1* mRNA, whereas *COX2* and *15S* rRNA levels remained unchanged, demonstrating a specific effect of Pet309 overexpression on the *COX1* mRNA accumulation (Fig. 5A,B). These data indicated that deletion of the last four PPR motifs in Pet309 does not abolish its interaction with the *COX1* mRNA. In contrast, overexpression of *pet309*Δ*nt-3xHA* failed to accumulate *COX1* mRNA (Fig. 5C,D). The inability of Pet309Δnt–3xHA to bind and stabilize the *COX1* mRNA is not due to alterations in its mitochondrial localization and association with the mitochondrial inner membrane, which is like the wild-type Pet309–3xHA (Fig. S2).

Overall, this data indicates that the first six PPR motifs of Pet309 are crucial for binding to the *COX1* mRNA and interacting with the mitoribosome, whereas the central and C-terminal end modules are not required for these processes. However, the three modules are indispensable for translational activity of Pet309.

### Mss51 interacts with the mitoribosome independently of the presence of Pet309 or the *COX1* mRNA

**Fig. 6.**
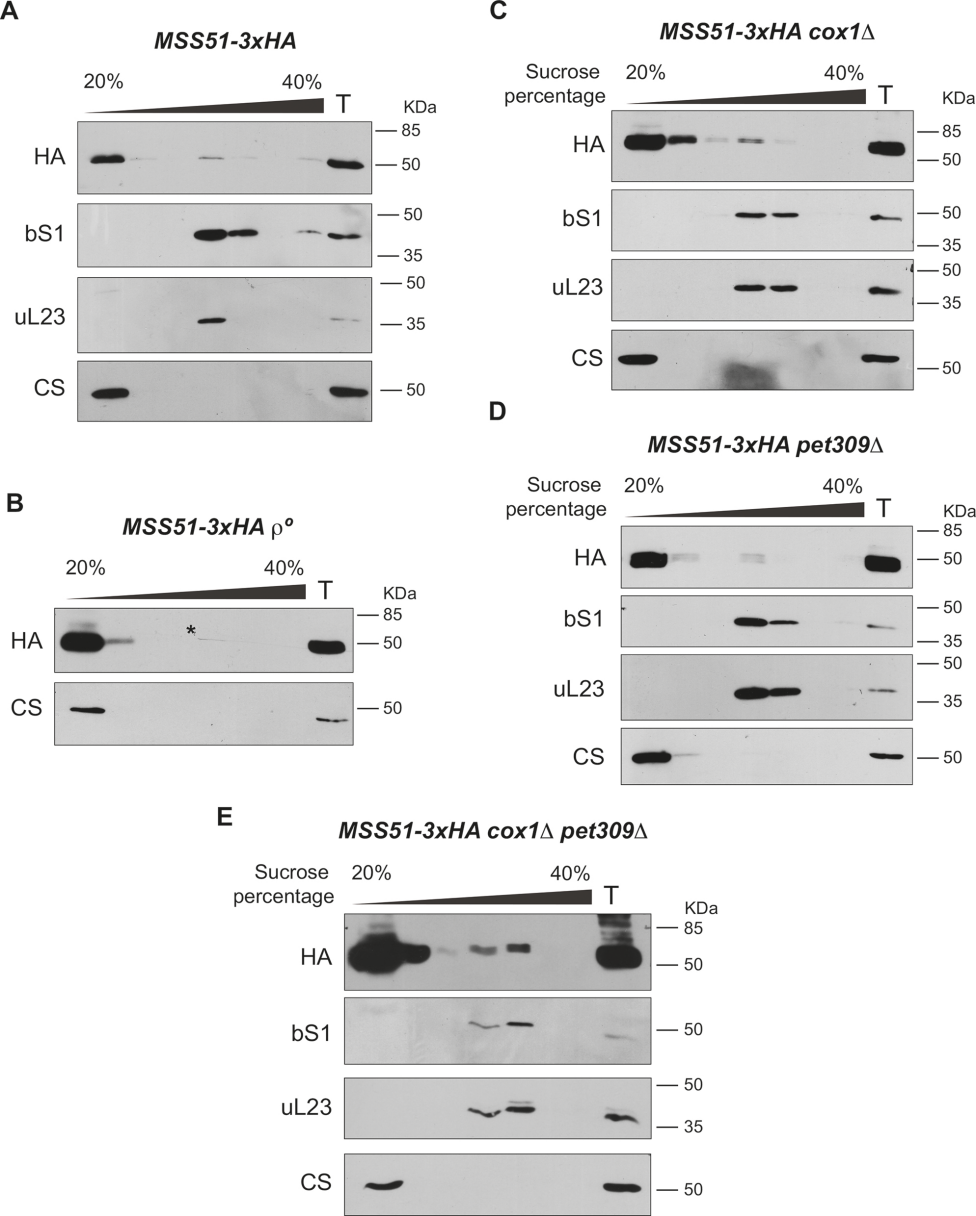
**Mss51 constitutively interacts with the mitoribosome.** Mitochondria from *MSS51-3xHA* cells carrying (A) WT mitochondrial DNA or (B) devoid of mtDNA (*ρ°*) were solubilized and separated by ultracentrifugation on a sucrose gradient. The resultant seven fractions from this gradient were analyzed by western blotting with the indicated antibodies. Similar experiments were carried out with mitochondria bearing (C) a deletion of the *COX1* gene (*cox1Δ*), (D) the mutant *PET309* gene (*pet309Δ*) and (E) the double mutant *cox1Δ pet309Δ*. For all experiments we used *n*=3 biological replicates. Uncropped blots are shown in Fig. S7. *Scratch on the original film, not a western blot signal.

Mss51 coordinates Cox1 synthesis and assembly by acting on the *COX1* 5′ UTR and physically interacting with the Cox1 peptide (Barrientos et al., 2004; Perez-Martinez et al., 2003, 2009). However, the mechanisms by which Mss51 activates *COX1* mRNA translation are yet to be understood. It has been previously reported that a population of Mss51 co-migrated in sucrose gradient centrifugations with the translational machinery (De Silva et al., 2017; Fontanesi et al., 2010; Mays et al., 2019; Verma et al., 2021). However, these co-migrations were mainly observed in C*c*O assembly mutants (De Silva et al., 2017; Fontanesi et al., 2010; Mays et al., 2019; Verma et al., 2021). Moreover, Mss51 was undetectable in the MIOREX complexes (Kehrein et al., 2015). Therefore, Mss51 association with the mitoribosome under wild-type conditions remains unclear. To illuminate the role of Mss51 as a translational activator, we focused on evaluating whether Mss51 associates with the mitoribosome in a wild-type context. We used a strain expressing Mss51–3xHA endogenously in its original locus, which was previously shown to not disturb its respiratory growth (Perez-Martinez et al., 2003). As expected, most of the Mss51–3xHA population was found in surface fractions of the sucrose gradient, but a small amount was detected co-migrating with the mitoribosome (Fig. 6A). In contrast, in a *ρ°* strain lacking mitochondrial DNA (and thereof mitoribosomes), Mss51 was solely detected in surface fractions, indicating that a small fraction of Mss51 is associated with mitoribosomes (Fig. 6B). Given that only a small population of Mss51 co-migrated with the mitoribosome, it is possible that the Mss51–mitoribosome interaction could be transient and occur only when the *COX1* mRNA is being translated. To determine whether Mss51 association with the ribosome depends on active translation of the *COX1* mRNA, we analyzed Mss51–3xHA migration in the *cox1*Δ strain used earlier (Fig. 2A). Interestingly, Mss51–3xHA co-migrated in sucrose gradients with bS1 and uL32, indicating that Mss51 interacted with the mitoribosome independently of the presence of the *COX1* mRNA (Fig. 6C). Previous reports have demonstrated that Pet309 and Mss51 physically interact (Zamudio-Ochoa et al., 2014), and as we found that Pet309 constitutively interacts with the mitoribosome, we hypothesized that the Mss51–mitoribosome interaction could be mediated by Pet309. To address this hypothesis, we analyzed the association of Mss51 with the mitoribosome in a mutant lacking Pet309. Mss51–3xHA maintained its co-migration with bS1 and uL23 in the absence of Pet309 (Fig. 6D), and the same result was obtained after simultaneous deletion of Pet309 and the *COX1* gene (Fig. 6E). Taken together, these results indicate that a small fraction of Mss51 constitutively interacts with the mitoribosome, independently of the presence of the *COX1* mRNA and Pet309.

## DISCUSSION

### Pet309 as a constitutive interactor of the mitoribosome

Pet309 and Mss51 were first described 30 years ago (Decoster et al., 1990; Manthey and McEwen, 1995), yet their mechanisms of translational activation remain unclear. In this study, we demonstrated that Pet309 and Mss51 interact with the mitoribosome independently of each other and of the *COX1* mRNA. Whereas Pet309 was not detected as a ribosome-free protein, only a minor proportion of Mss51 interacted with the mitoribosome. This aligns with previous findings, as Mss51 also interacts with Cox1 assembly intermediates (Fontanesi et al., 2011; Mick et al., 2010; Perez-Martinez et al., 2003; Pierrel et al., 2007).

The instability of Pet309 in the absence of mitoribosomes (in the *ρ°* strain) suggests a constitutive association of Pet309 with the translational machinery. This decrease in steady state levels of Pet309 is unlikely due to changes in its synthesis, as cytosolic translation is unaffected in *ρ°* strains (Couvillion et al., 2016). Mitoribosomal proteins are prone to degradation when the ribosome is not assembled (Graack and Wittmann-Liebold, 1998), a phenomenon we also observed for bS1 and uL23 in our *ρ°* strains. Our findings suggest that Pet309 is more than a transiently associated ribosome-accessory protein; it is constitutively associated to the mitoribosome. This is supported by the observation that the association of Pet309 with the mitoribosome in MIOREX complexes is resistant to high ionic strength, whereas the helicase Mss116 dissociates under similar conditions (Kehrein et al., 2015).

Intriguingly, Pet309 can interact with the mitoribosome even in the absence of Mss51 or the *COX1* mRNA, although its stability is compromised if either is missing. In fact, the combined absence of both Mss51 and *COX1* mRNA further destabilizes Pet309. This might suggest that a complete *COX1* mRNA translation machinery is necessary to protect Pet309 from degradation.

The association of Pet309 with the mitoribosome occurs independently of the *COX1* mRNA, implying that certain mitoribosomes might be preloaded with Pet309 to initiate *COX1* mRNA translation. This raises the question of whether Pet309 is a marker of specialized ribosomes dedicated to Cox1 synthesis or mitochondria-encoded C*c*O subunits. The concept of heterogeneous ribosome populations preferentially translating specific mRNAs is gaining acceptance (for a review, see Guo, 2018). Indeed, the yeast mitoribosome cryo-electron microscopy structure reveals a non-identified protuberance above the mRNA exit tunnel, which might represent different translational activators that label a subset of mitoribosomes (Desai et al., 2017).

Pet309 is composed almost entirely of PPR motifs. We previously demonstrated that eliminating a single PPR motif in the central module rendered a non-functional protein, although it could still bind *COX1* mRNA (Tavares-Carreón et al., 2008). Although the target sequence of Pet309 has not been identified, two sites – at −356 to −335 and −76 to −55 nt from the *COX1* mRNA AUG start codon – are predicted as binding sites (De Silva et al., 2017). Deletion of a single PPR or multiple repeats, as in this study, likely disrupts the RNA–protein recognition code. AlphaFold predicts ∼24 PPR motifs in Pet309, forming three distinct modules. The central module, composed of 12 PPR motifs, is the most structured, forming a compact superhelical structure. The N-terminal module, containing six PPR motifs, is predicted to have a more open, planar structure, whereas the C-terminal end module consists of three well-structured PPR motifs followed by three disorganized PPR motifs, with varying α-helix lengths and turns within some helices (Fig. 3A; Fig. S1A). We demonstrated that the central and C-terminal end modules of Pet309 are dispensable for its binding to *COX1* mRNA and the mitoribosome, whereas the N-terminal module is essential for both. Although Pet309Δnt–HA lacks association with the mitoribosome, it was detected in the second fraction of the gradient, suggesting its presence in a complex with other proteins, possibly protecting it from degradation, and a 900-kDa complex containing Pet309 has been described previously (Krause et al., 2004), which includes the *COB* mRNA translational activator Cbp1 (Islas-Osuna et al., 2002; Mittelmeier and Dieckmann, 1990).

Deleting the N-terminal module of Pet309 also abolished its interaction with the *COX1* mRNA. The ribosomal location of Pet309 might be essential for *COX1* mRNA interaction, supported by findings that the *COX1* mRNA associates with the mitoribosome even in the absence of Pet309 (De Silva et al., 2017), and that mitochondrial mRNAs are channeled from transcription to translation by specialized factors (Kehrein et al., 2015). Nam1, which associates with mitoribosomes and interacts with RNA polymerase and various translational activators, including Pet309, might be one such channeling factor (Bryan et al., 2002; Kehrein et al., 2015; Markov et al., 2009; Naithani et al., 2003; Rodeheffer et al., 2001). We believe the lack of interaction between Pet309Δnt and the *COX1* mRNA is due to the altered location of mutant, distant from translation machinery and channeling factors.

Beyond the initial six PPR motifs, other PPR mutations analyzed in this and prior studies did not affect the ability of Pet309 to bind the *COX1* mRNA and the mitoribosome, although they did impair Cox1 synthesis (Zamudio-Ochoa et al., 2014). Two potential explanations exist – first, that missing PPR motifs disrupt the ability of Pet309 to accurately recognize the *COX1* sequence, mispositioning the mitoribosome; or second, that these deletions affect the interaction of Pet309 with other proteins. The DEAD-box helicase Mss116 interacts with Pet309 and is proposed to unwind *COX1* secondary structures to facilitate sequence-specific binding by Pet309. Interestingly, Mss116 mutants affecting helicase activity do not disrupt its interaction with Pet309, although *COX1* mRNA translation is impaired (De Silva et al., 2017).

### Pet309 and Mss51 role in activating *COX1* mRNA translation

**Fig. 7.**
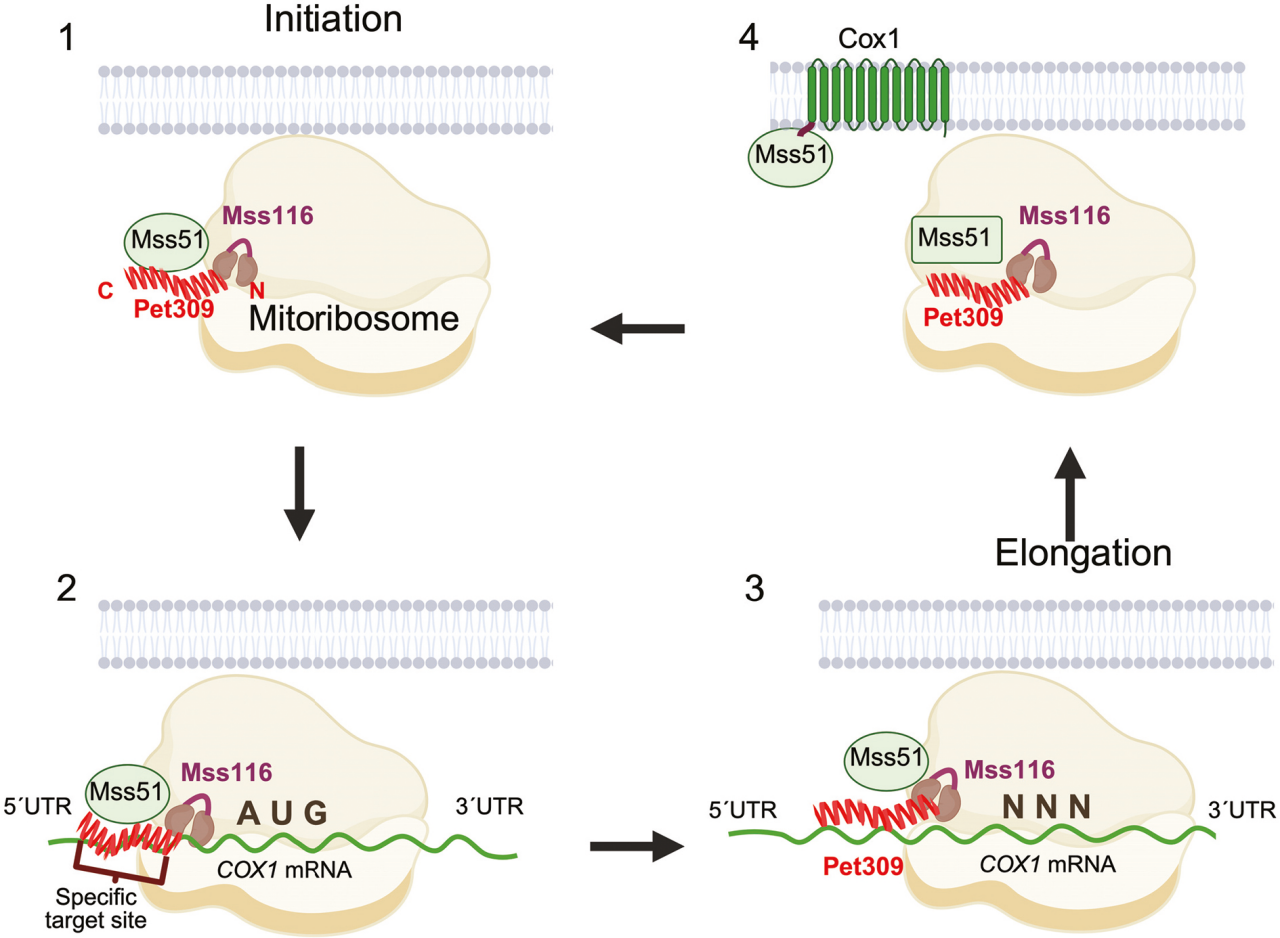
**Model for the mechanisms of Pet309 and Mss51 on translation of the *COX1* mRNA.** See text in Discussion section for details. Created in BioRender by Perez, X., 2025. https://BioRender.com/jqiwlc8. This figure was sublicensed under CC-BY 4.0 terms.

Mss51 is another essential translational activator of *COX1* mRNA, but unlike Pet309, it plays a dynamic role in C*c*O biogenesis by coordinating Cox1 synthesis and assembly (Barrientos et al., 2004; Mick et al., 2010; Perez-Martinez et al., 2009). In this study, we found that Mss51 interacts with the mitoribosome, although this interaction appears weak and/or transient, as only a minor fraction of Mss51 was detected in ribosomal fractions and it was absent in MIOREX complexes (Kehrein et al., 2015). Interestingly, Mss51 remains associated with the mitoribosome even in the absence of the *COX1* mRNA, suggesting that, a subset of Mss51 is constitutively associated with the translational machinery independently of *COX1* mRNA translation. This indicates that Mss51, like Pet309, might interact with the mitoribosome prior to Cox1 synthesis. Both Pet309 and Mss51 activate *COX1* mRNA translation, but their interactions with the mitoribosomes are independent. Based on these results and prior studies on Cox1 biogenesis, we propose the following model for the *COX1* mRNA translation (Fig. 7).

First, there is a preloading phase where Pet309, Mss51 and Mss116 associate with the mitoribosome before the *COX1* mRNA is loaded*.* Our data suggest that all of Pet309 is bound to the mitoribosome, whereas Mss51 exists in two populations, one engaged in Cox1 assembly and a smaller fraction associated with the mitoribosome. Pet309 is constitutively associated to the mitoribosome, as it withstands high ionic strength conditions, via its N-terminal module, whereas Mss116 acts as a ribosomal accessory, detaching under similar conditions (Kehrein et al., 2015).

Second, mRNA binding and positioning occurs, where the helicase Mss116 unwinds the secondary structure of the approaching *COX1* mRNA to enable Pet309 binding. Mss51 induces a conformational change on Pet309 enabling it to recognize its specific sequence on the 5′ UTR and to position the mitoribosome active site on the AUG start codon. Two putative sequences on the *COX1* 5′UTR could be targets of Pet309 (De Silva et al., 2017). The helicase Mss116 assists the binding process, probably by unwinding secondary structures in the mRNA to facilitate Pet309 to localize its cognate sequence (De Silva et al., 2017). However, these interactions alone might be insufficient for accurate Pet309 sequence binding and correct ribosome positioning, as seen in *mss51*-null mutants that exhibit abnormal *COX1* mRNA translation dependent on Pet309. (Zambrano et al., 2007). Mss51 could facilitate this process by altering the conformation of Pet309, modifying the interaction of Pet309 with the *COX1* 5′ UTR*.* This aligns with previous findings showing that IP of Pet309 is inefficient without Mss51, likely due to conformational changes affecting epitope accessibility, although Pet309 still interacts with the *COX1* mRNA (Zamudio-Ochoa et al., 2014). Indeed, PPR proteins frequently undergo conformational shifts (Ban et al., 2013; Ke et al., 2013; Shen et al., 2016; Yin et al., 2013).

The third step is translation elongation. Here, Pet309 remains bound to the mitoribosome throughout translation but does not actively participate in elongation, whereas Mss51 and Mss116 are still required. Evidence shows that Pet309 binds to the *COX1* mRNA even when elongation is inhibited by puromycin (Zamudio-Ochoa et al., 2014). Although Mss51 and the helicase Mss116 target additional *COX1*-coding sequence regions, Pet309 acts exclusively on the 5′ UTR (De Silva et al., 2017; Perez-Martinez et al., 2003, 2009; Zamudio-Ochoa et al., 2014).

The final step is post-translation Cox1 assembly. After *COX1* mRNA translation, Pet309 remains attached to the ribosome, whereas Mss51 engages in the assembly of the nascent Cox1 peptide, where it might change its own conformation and/or association with the ribosome (Barrientos et al., 2004; Mick et al., 2010; Perez-Martinez et al., 2003). At least three different Mss51 conformations have been proposed (Fontanesi et al., 2011; Mick et al., 2011, 2010), although it is unclear which conformation remains ribosome-bound or whether additional conformations exist. After assisting with Cox1 assembly, Mss51 adopts a translationally inactive conformation until new rounds of translation initiation take place (step 1).

Many questions remain to be answered in future studies, such as how does Pet309 specifically recognize the *COX1* mRNA among other mitochondrial mRNAs, how do Pet309, Mss51 and Mss116 associate to the ribosome during translation termination and ribosome recycling, and what exact role does Mss51 play in aiding Pet309 and the ribosome to locate the *AUG* start codon. Also it needs to be determined whether there are similarities between yeast Mss51 and the mechanisms of its mammalian counterparts.

## MATERIALS AND METHODS

### Yeast strains, media and genetic methods

The *S*. *cerevisiae* strains used in this study are listed in Table S1. Standard genetic methods and media recipes were as previously described (Burke et al., 2000). Complete fermentable media were YPD or YPRaf (containing 2% glucose or 2% raffinose). Minimal medium contained 0.67% yeast nitrogen base, 2% glucose or 3% ethanol/3% glycerol, and Complete Supplement Mixtures (CSMs) purchased from Bio 101 (Vista, CA, USA) and ForMedium (UK). Gene deletion constructs with *KANMX4*, *LEU2* or *URA3* cassettes were generated by PCR.

### Mitochondrial transformation, and integration of the *cox1Δ* construct into *ρ*^+^ mtDNA

A plasmid containing the *COX1* gene, including 1365 bp upstream of the 5′ UTR and 990 bp of the 3′ UTR was digested with *Pac1* and re-ligated to create a deletion of the complete *COX1* ORF, together with 787 bp of the 5′ UTR and 525 bp of the 3′ UTR, to create plasmid pXPM75. A fragment of the *COX2* gene was amplified by PCR and cloned into the *ClaI-HindIII* sites in pXPM75 to create plasmid pCB14. This plasmid was transformed on Nab69 *ρ°* by high-velocity microprojectile bombardment (Camacho-Villasana et al., 2024)**.** Mitochondrial transformants were identified by their ability to rescue respiratory growth when mated to a *ρ^+^* strain bearing the A114 to stop mutation in the mitochondrial *cox2* gene. The *cox1Δ* construct was integrated by homologous recombination into *ρ^+^* mtDNA by isolating cytoductants issued from crosses of the transformant to strain TF258 (Perez-Martinez et al., 2009).

### Cloning of the pet309Δnt–3xHA and pet309Δct–3xHA constructs

The plasmids used and generated during this work are derivatives of pXP96, pXP97 and pXP104 (Tavares-Carreón et al., 2008), and contain the *PET309-3xHA* sequence, including 310 and 205 nt of the *PET309* 5′ and 3′ UTRs. Pet309 mutants were constructed similarly to previously reported *(*Zamudio-Ochoa et al., 2014*)*. *PET309*Δ*nt* lacks residues A53 to Q311, and *PET309*Δ*ct* lacks residues L760 to V962. Both mutants were generated by fusion PCR (Ho et al., 1989). Site-directed mutagenesis was undertaken by overlap extension using PCR, using Accuzyme DNA polymerase (Bioline) and pXP97 as the DNA template. *PstI/EcoRI*-digested PCR products were cloned into similarly digested pXP96. The *XbaI*–*XhoI* DNA fragments were ligated into pXP97 to generate the ARS/CEN, low-copy-number plasmids. For high-copy-number plasmids, the *XbaI-ClaI* fragments from mutant-bearing pXP96 plasmids were ligated into the pXP104 plasmid.

### Sucrose fractionation of mitochondrial lysates

This protocol was as previously reported (García-Guerrero et al., 2018). Briefly, a sample of 500 µg of mitochondrial protein was incubated with lysis buffer (1% digitonin, 10 mM MgOAc, 50 mM NaCl, 20 mM HEPES-KOH pH 7.4 and 1 mM PMSF) for 30 min on ice. The mitochondrial lysate was clarified by centrifugation at 16,200 ***g*** (13,500 rpm in a benchtop centrifuge) for 10 min. The supernatant of the centrifugation was then loaded into a discontinuous gradient of 40, 30, and 20% sucrose containing 0.1% digitonin, 10 mM MgOAc_2_, 20 mM DTT, 10 mM Tris-HCl, pH 7.4, and 0.5 mM PMSF. The sucrose gradients were ultracentrifuged at 145,000 ***g*** (39,000 rpm) in a SW-55Ti rotor for 2 h at 4°C. Fractions of 600 μl were TCA-treated for protein precipitation. Proteins were resolved by SDS-PAGE, transferred to PVDF membranes, and detected by immunoblotting with the indicated antibodies.

### RNA immunoprecipitation assay

This protocol was done as previously reported (Zamudio-Ochoa et al., 2014). Briefly, 1 mg of mitochondrial protein was lysed with 500 μl of 0.7% n-dodecyl β-D-maltoside, 100 mM NaCl, 20 mM Tris-HCl pH 7.4, 200 U of RNaseOUT (Invitrogen), and protease inhibitors (Roche). The cleared lysate was incubated with an anti-HA high-affinity antibody (Roche) coupled to protein A–Sepharose (GE Healthcare). The immunoprecipitate was washed and a quarter of the supernatant and precipitate fractions were saved for western blot analysis. The remainder was used for RNA extraction with TRIzol reagent. 20 ng of RNA were treated with 1 unit of DNase I (Invitrogen) for 15 min at 25°C. The cDNA was prepared by addition of primers for *COX1* or *VAR1* in the presence of SuperScript III Reverse Transcriptase (Invitrogen). This cDNA was used as template for PCR reactions to amplify the *COX1* or *VAR1* 5′ UTRs. For a primer list, see Table S2.

### Northern blotting

Total RNA was extracted from yeast cells using the RNeasy Mini kit (Qiagen). 10 µg of total RNA was separated by denaturing agarose gel electrophoresis and transferred to Hybond XL membrane (GE Healthcare). Membranes were probed with radioactively labeled probes recognizing *COX1* exon 4, *COX2* and *15S* rRNA. Blots were analyzed with a Typhoon 8600 PhosphorImager (GE Healthcare) and quantified with ImageQuaNT. For statistical analysis, a Log2 fold change of the *COX1*-to-*15S* ratio was determined relative to the control (wild-type Pet309 in low copy number plasmid). These data were used to perform a one-way ANOVA using the Geisser–Greenhouse correction, followed by a Tukey's multiple comparisons test.

### Membrane protein analyses

For membrane protein analysis, 100 µg of purified mitochondria was resuspended in 400 μl of 0.1 M Na_2_CO_3_, vortexed and incubated on ice for 60 min. The sample was centrifuged at 100,000 ***g*** (65,000 rpm on a TLA-100.3 rotor) for 10 min, and the supernatant was precipitated with 10% trichloroacetic acid.

For separation of mitochondria in membrane and soluble fractions, 100 µg of mitochondria was resuspended in 100 mM NaCl 100 mM, HEPES 20 mM and sonicated. The samples were centrifuged at 100,000 ***g*** (65,000 rpm on a TLA-100.3 rotor) for 10 min, and the supernatant was precipitated with 10% trichloroacetic acid. Antibodies were anti-HA conjugated to HRP (1:2000, Roche 12013819001), anti-HA (5 μl in 50 μl of protein A Sepharose beads, Roche 11867423001) and anti-citrate synthase, anti-bS1, anti Yme1 and anti-Arg8 (all prepared on rabbit and used at 1:1000, 1:2000, 1:1000 and 1:10,000, respectively; kind gifts from Thomas D. Fox, Cornell University, USA). Rabbit anti uL23, anti-cytochrome *c_1_* and anti-Cyt*b* were kind gifts from Rosemary Stuart (Marquette University, Milwaukee, WI, USA) and were used at 1:20,000, 1:1000 and 1:1000, respectively, polyclonal rabbit anti-Cox1 (1:5000) was prepared in our laboratory. Secondary antibodies were goat anti rabbit-IgG conjugated to HRP (1:5000; Jackson ImmunoResearch, 111-035-003) and goat anti-mouse-IgG (1:5000; Santa Cruz Biotechnology, sc-2005).

### Proteinase K digestion of mitochondria

Samples of 100 µg of purified mitochondria were suspended in HEPES-KOH 20 mM pH 7.4 with or without sorbitol 0.6 M. The samples were incubated on ice with 100 μg/ml of proteinase K. Digestion was terminated with 2 mM phenylmethylsulfonyl fluoride.

### Image digitalization and processing

Original films were scanned, and edited brightness and contrast by Adobe Photoshop software following all scientific ethical criteria. Original scans are shown in Figs S4–S9.

### AI tool use

The ChatGPT application was used for proofreading, followed by careful review from all authors.

## Supplementary Material



10.1242/joces.263694_sup1 Supplementary information
