# Supplementary material for: Insights into the translational activation mechanisms of the COX1 mRNA in yeast mitochondria
Source: J Cell Sci. 2025 Sep 2;138(16):jcs263694. doi: 10.1242/jcs.263694 (PMC12450462; doi:10.1242/jcs.263694)
Supplement: Supplementary information [file joces-138-263694-s1.pdf]

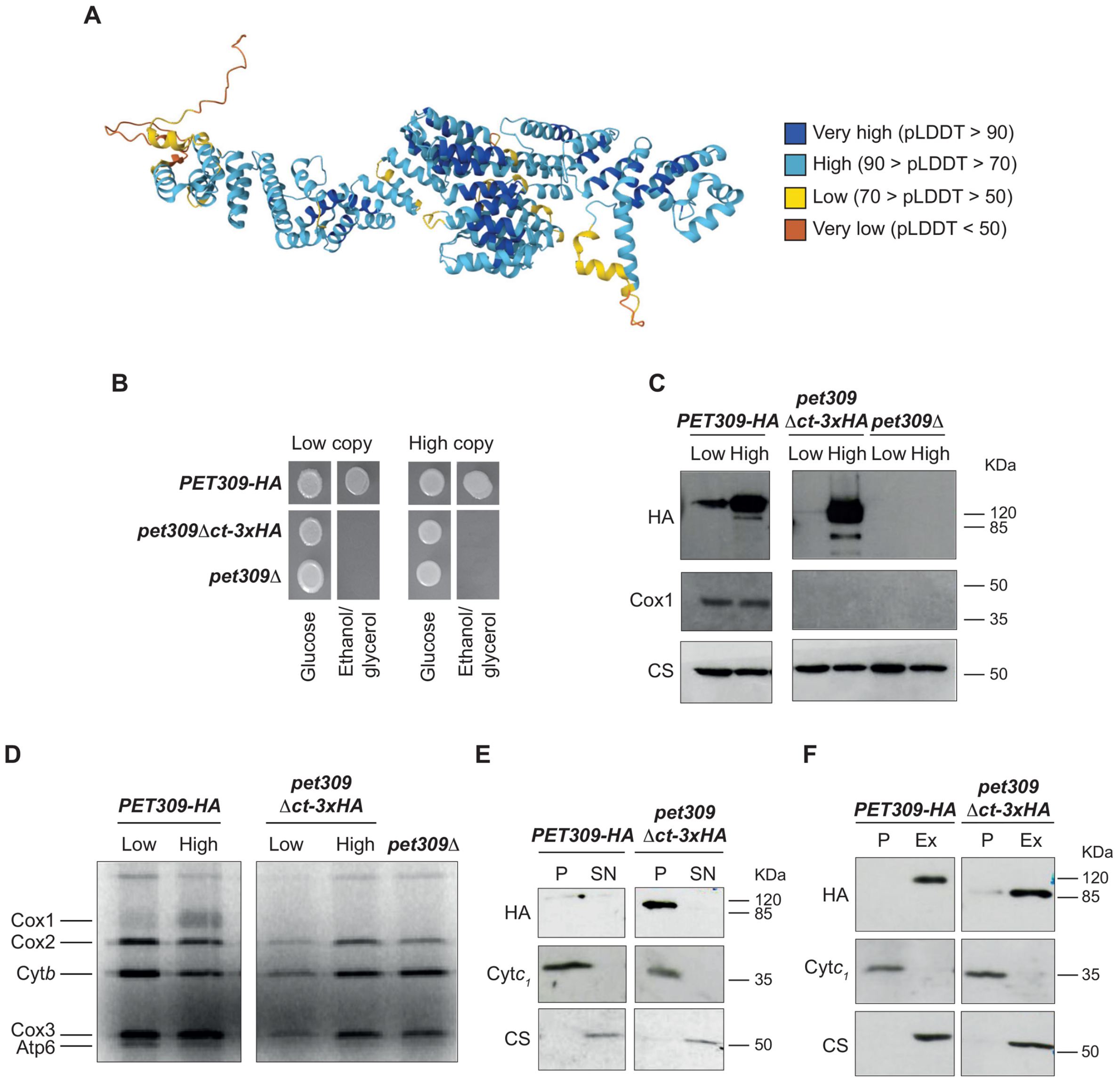

Fig. S1. A mutant lacking the C-terminus module of Pet309 localized to mitochondria like a wild type Pet309 protein. A) The Pet309 full sequence was analyzed in the AlphaFold software. The model is shown with the color code used by the program to indicate values of confidence. The model obtained a pTM value of 0.73 (Jumper et al., 2021). B) The *pet309Δct-3xHA* and the *PET309-3xHA* constructs were cloned in either centromeric (Low) or 2 µ (High) yeast expression plasmids and were transformed in cells lacking the endogenous *PET309* gene (pet309Δ was transformed with empty plasmid, as negative control). Growth on fermentative or respiratory media was tested for 3 days at 30°C. C) The same strains were used for western blot analysis of mitochondria using the indicated

Journal of Cell Science • Supplementary information

antibodies. D) Cells were incubated with ^{35}S-methionine and cycloheximide to inhibit cytoplasmic ribosomes. Mitochondrial translation products were analyzed by SDS-PAGE and autoradiography. E) Purified mitochondria from *PET309-3xHA* or *pet309Δct-3xHA* strain were sonicated to break the membranes. The membrane (P) and the soluble fractions (SN) were separated by ultracentrifugation, resolved by SDS-PAGE and analyzed by Western blot. Anti-Cytc_1 antibody was used as a control of a membrane protein and anti-CS (citrate synthase) antibody was used as a control of a soluble protein. F) Purified mitochondria from *PET09-3xHA* and *pet309Δct-3xHA* strain were treated with Na_2CO_3 to extract non-integral proteins from the mitochondrial membranes. After ultracentrifugation, membranes (pellet, P) and extracted and soluble proteins (EX) were resolved with SDS-PAGE and analyzed by Western blot. The Anti-Cytc_1 antibody was used as a control of an integral membrane protein and the anti-CS antibody as a control of a soluble protein. Uncropped blots in Fig. S8.

Journal of Cell Science • Supplementary information

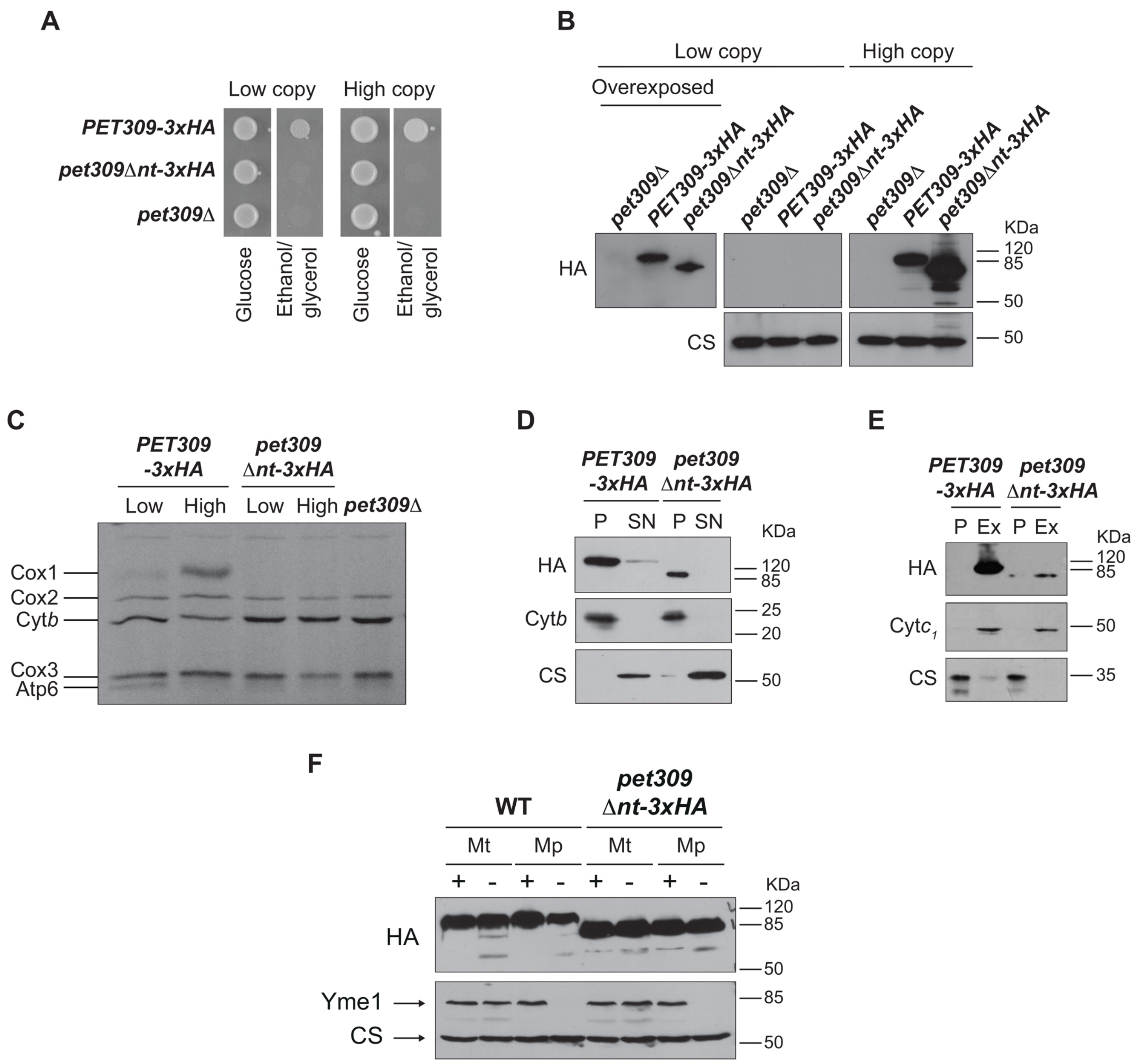

Journal of Cell Science • Supplementary information

Fig. S2. A mutant lacking the N-terminus module of Pet309 localized to mitochondria like a wild type Pet309 protein. A) The *pet309Δnt-3xHA* and the *PET309-3xHA* constructs were cloned in either centromeric (Low) or 2μ (High) yeast expression plasmids and transformed in cells lacking the endogenous *PET309* gene. *pet309Δ* strain was transformed with empty plasmid. Growth on fermentative or respiratory media of the indicated cells was tested for 3 days at 30°C. B) The same strains were used for western blot analysis of mitochondria. C) Cells were incubated with ^{35}S-methionine and cycloheximide as in Supplementary Figure S1. D) Purified mitochondria from *PET309-3xHA* or *pet309Δnt-3xHA* strain were sonicated to break the membranes. The membrane (P) and the soluble fractions (S) were separated by ultracentrifugation, and analyzed by Western blot as in Supplementary Figure S1. E) Purified mitochondria from Pet309-3xHA and *pet309Δnt-3xHA* strain were treated with Na_2CO_3 to extract non-integral proteins from the mitochondrial membranes. After ultracentrifugation, membranes (pellet, P) and extracted and soluble proteins (EX) were resolved with SDS-PAGE and analyzed by Western blot. F) Purified mitochondria from the *PET309-3xHA* or *pet309Δnt-3xHA* strains were incubated in hypotonic (Mitoplasts, Mp) or isotonic (Mitochondria, Mt) buffer in the absence (-) or presence (+) of Proteinase K. Samples were resolved by SDS-PAGE and analyzed by Western blot using anti-HA, anti-Yme1 and anti-Citrate synthase (CS) antibodies. Uncropped blots in Figure S8.

Journal of Cell Science • Supplementary information

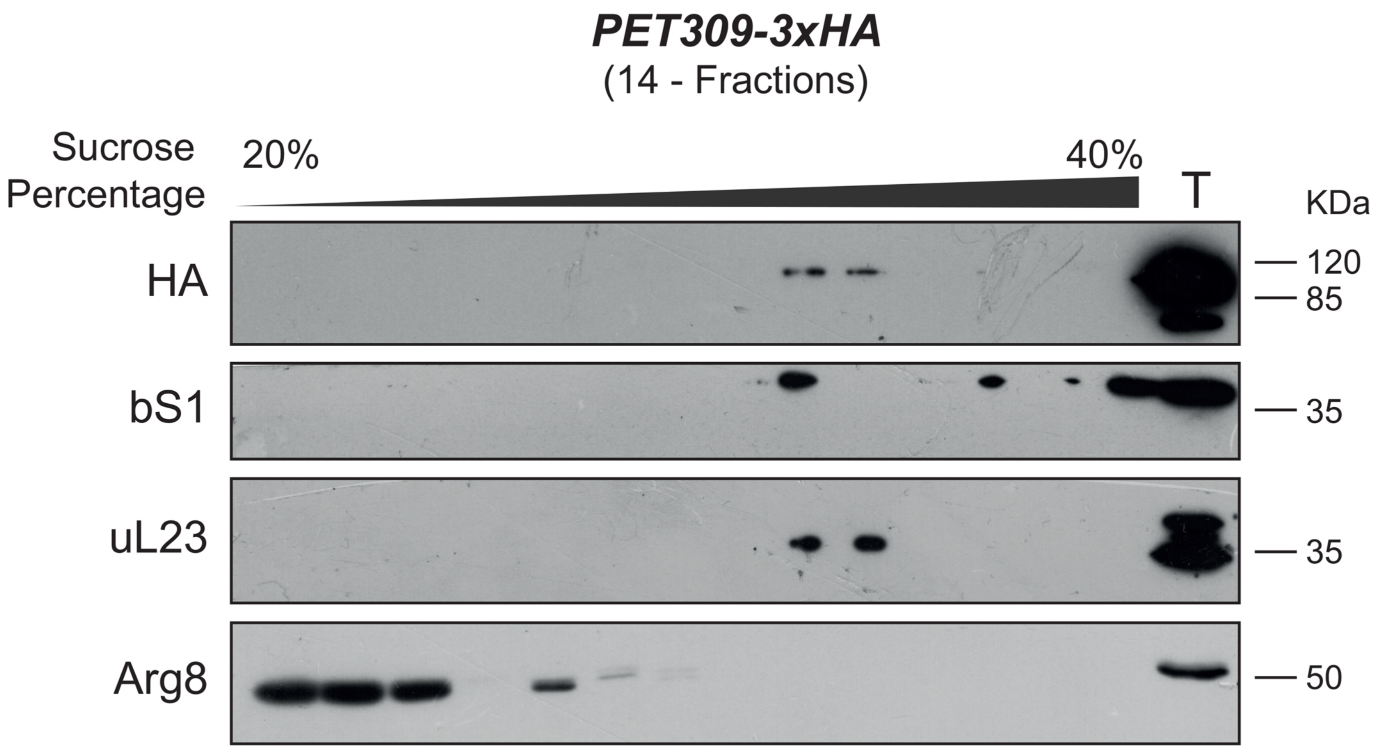

Fig. S3. Analysis of a sucrose ultracentrifugation gradient divided into 14 fractions. Western blot analysis of the sucrose gradient separation from mitochondria carrying the wt *PET309-3xHA* construct. bS1, small subunit protein. uL23, large subunit protein. CS, Citrate synthase. T, Total fraction, equivalent to 7% of the load. Uncropped blots in Figure S9.

Journal of Cell Science • Supplementary information

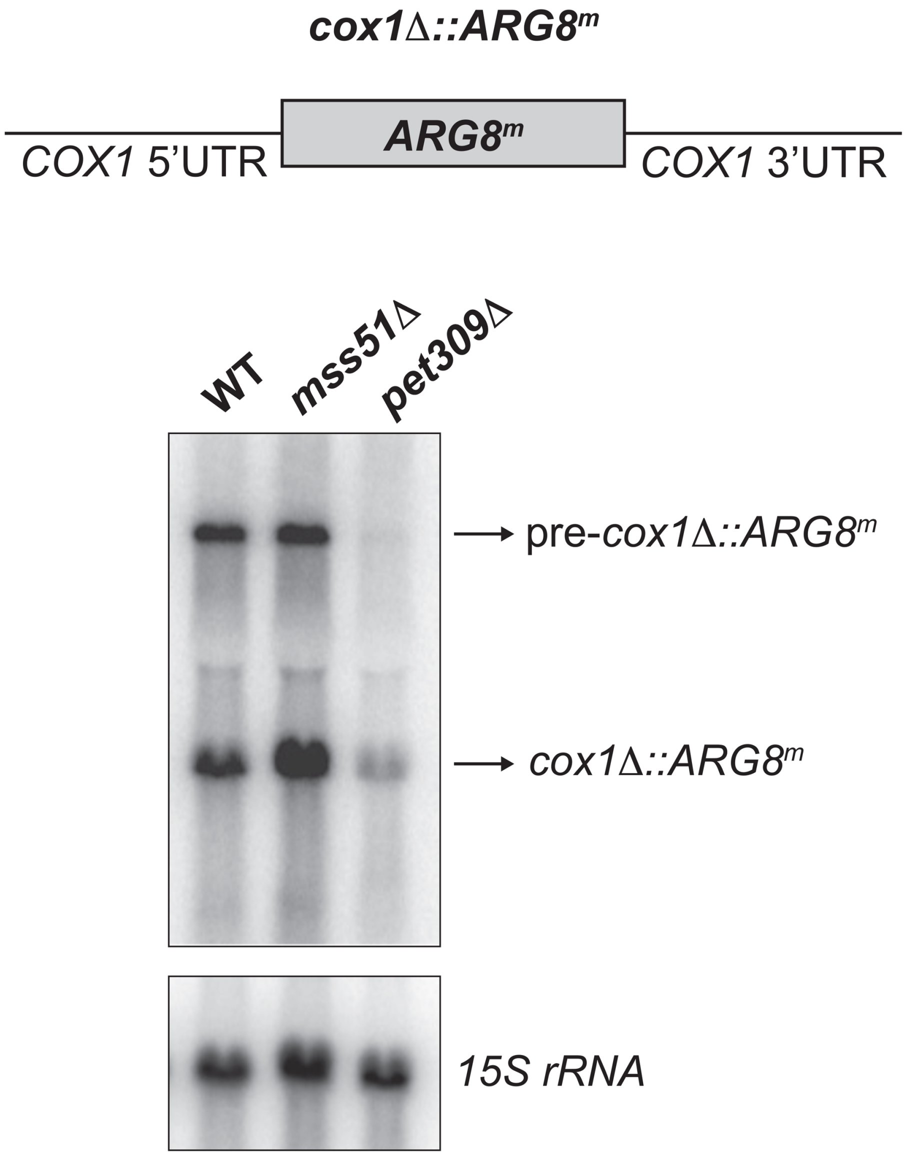

Fig. S4. Pet309, and not Mss51, is necessary for stability of the *cox1Δ::ARG8ᵐ* mitochondrial mRNA. 10 µg of total RNA from WT, *mss51Δ* or *pet309Δ* cells were isolated and separated by agarose electrophoresis. ^{32}P-radiolabeled probes for *ARG8ᵐ* and the 15S rRNA were used.

Journal of Cell Science • Supplementary information

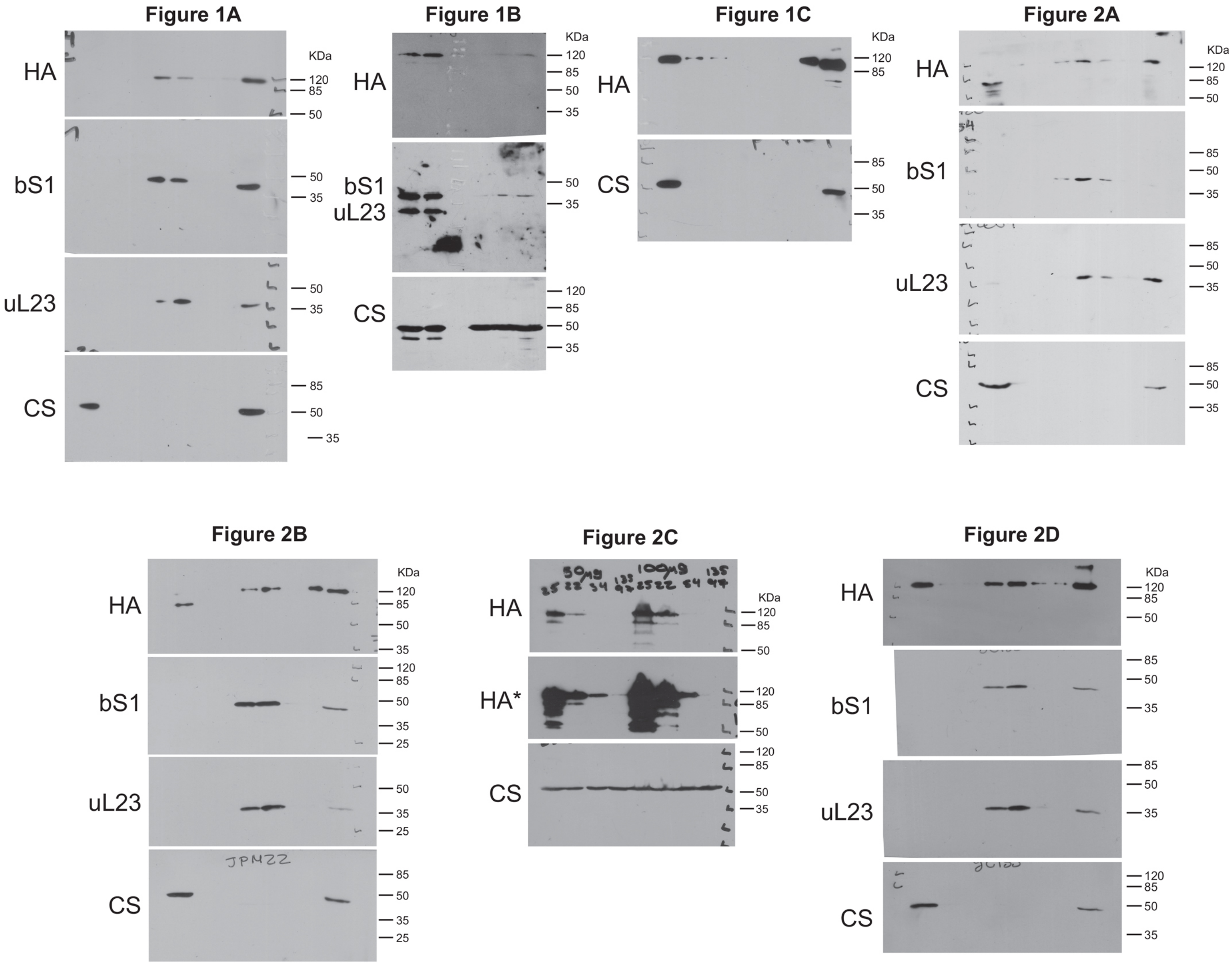

Fig. S5. Uncropped blots from Figs 1 and 2. Blots were imaged by film exposure and scanned for digitalization. Used antibodies indicated on each blot.

Journal of Cell Science • Supplementary information

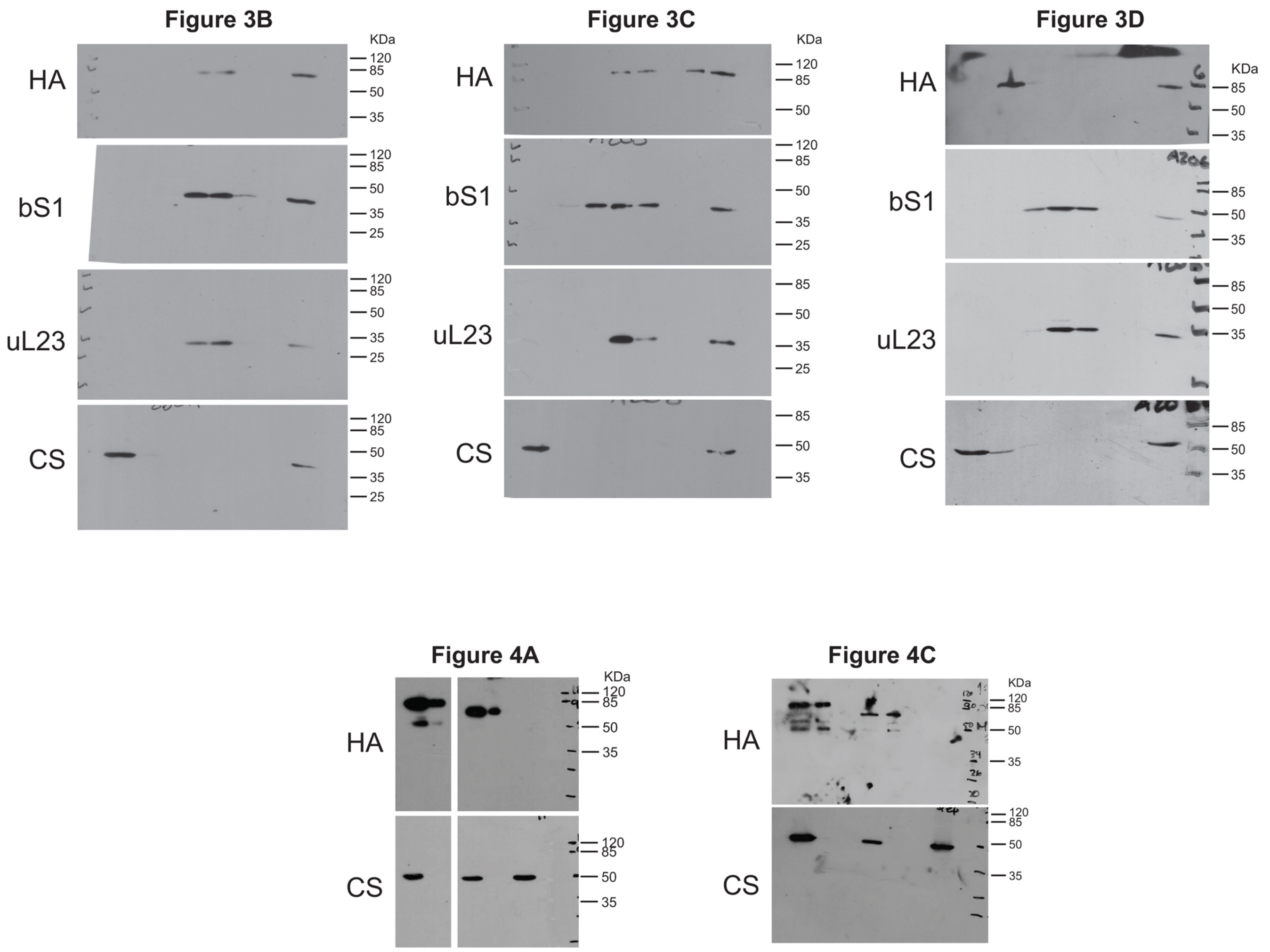

Fig. S6. Uncropped blots from Figs 3 and 4. Blots were imaged by film exposure and scanned for digitalization. Used antibodies indicated on each blot.

Journal of Cell Science • Supplementary information

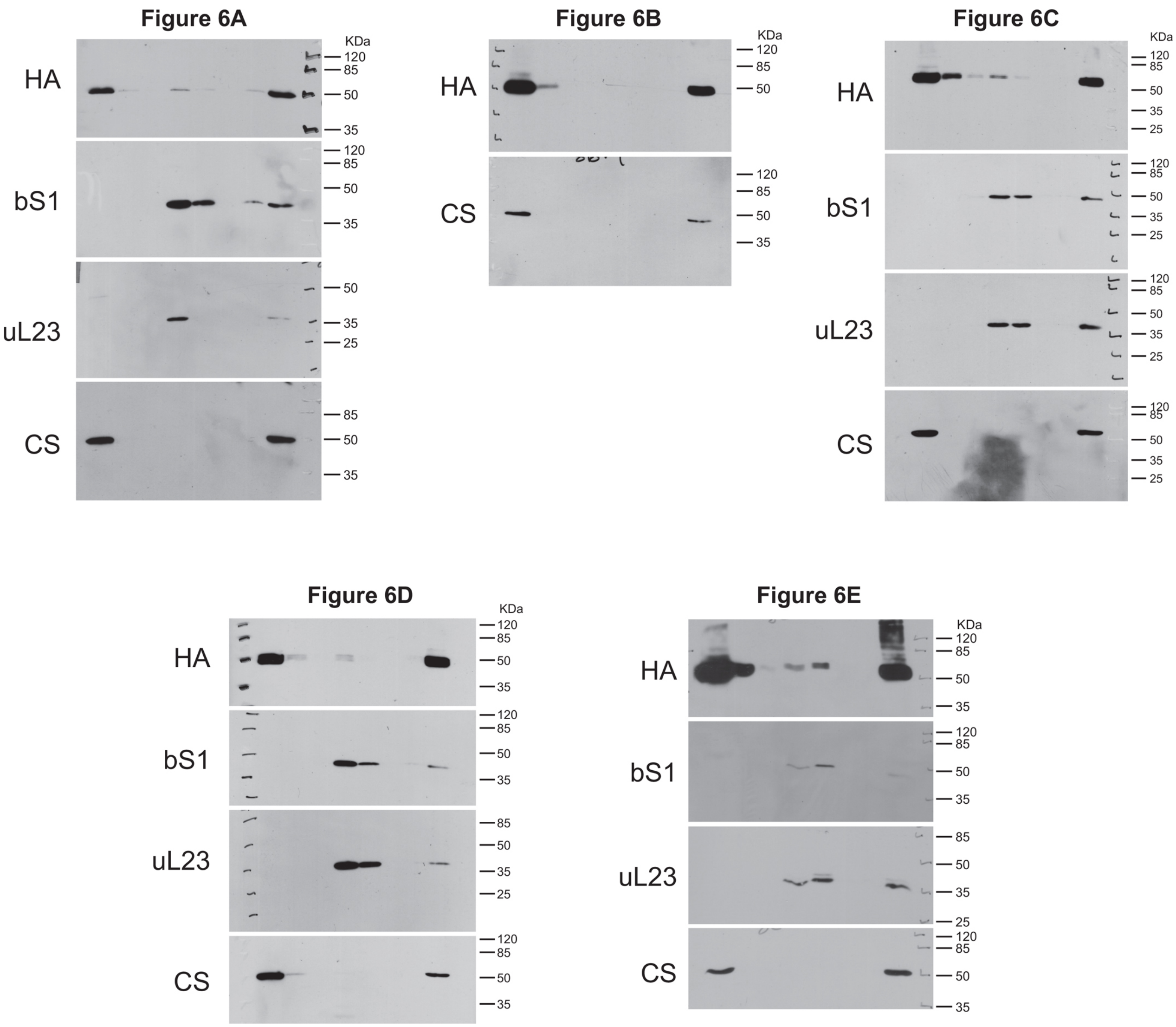

Fig. S7. Uncropped blots from Fig. 6. Blots were imaged by film exposure and scanned for digitalization. Used antibodies indicated on each blot.

Journal of Cell Science • Supplementary information

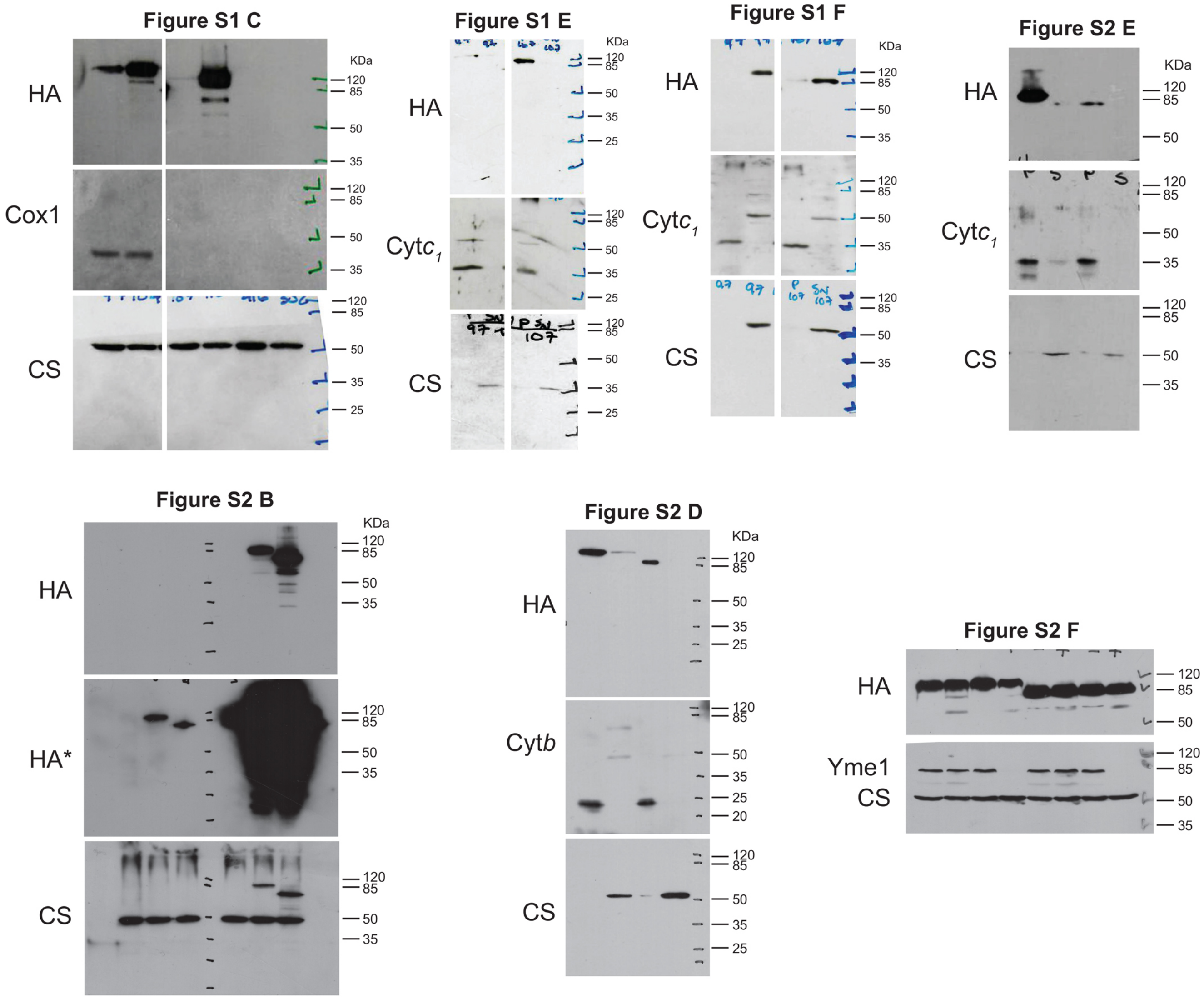

Fig. S8. Uncropped blots from Figs S1 and S2. Blots were imaged by film exposure and scanned for digitalization. Used antibodies indicated on each blot.

Journal of Cell Science • Supplementary information

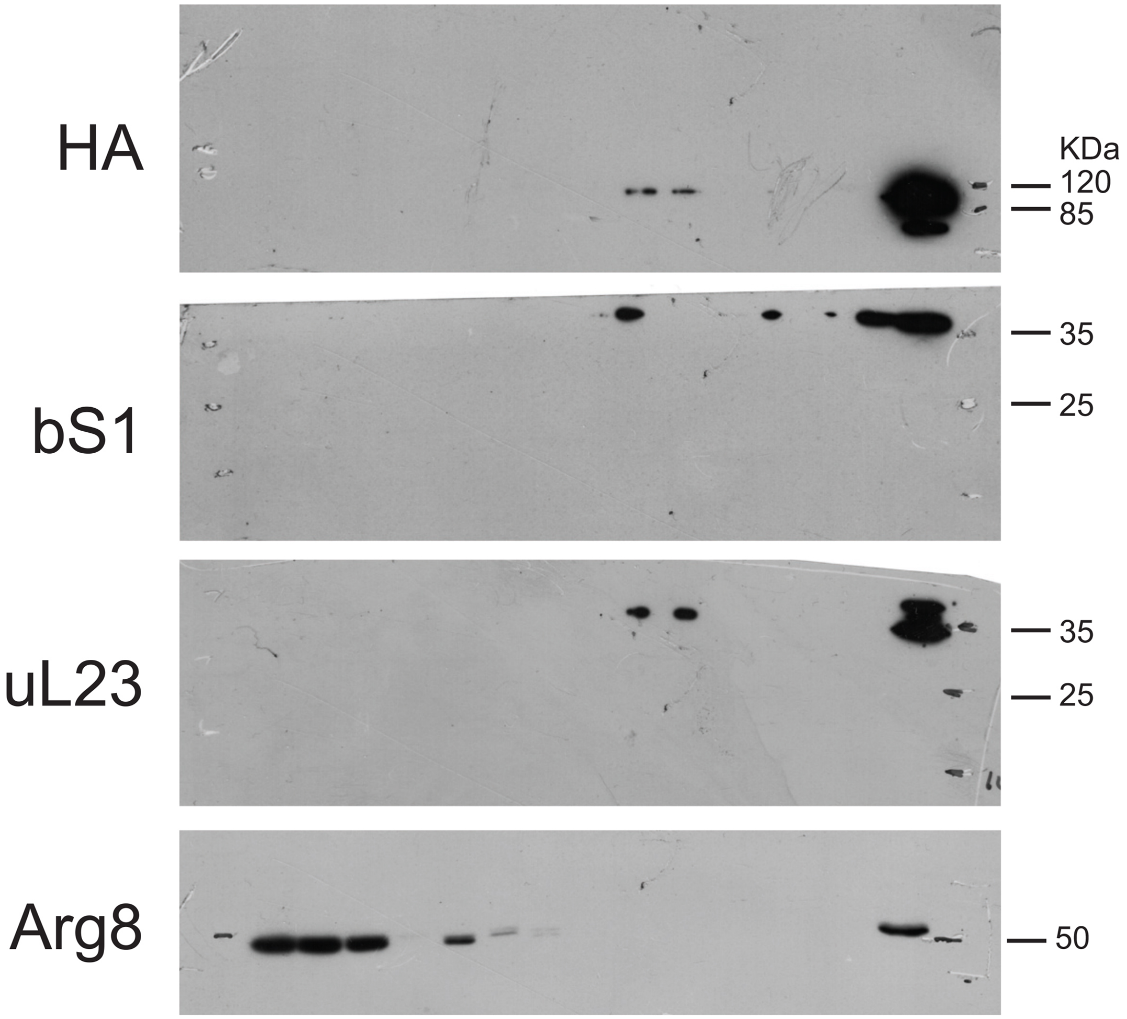

Fig. S9. Uncropped blots from Figs S3. Blots were imaged by film exposure and scanned for digitalization. Used antibodies indicated on each blot.

Journal of Cell Science • Supplementary information

Table S1. *S. cerevisiae* Strains Used in This Study

Strain	Genotype	Reference
NAB69	*Mata, ade2-101, arg8::hisG, ura3-52, kar1-1 [rho]*	(Bonnefoy &Fox, 2000)
AGG54	*Mata, ura3-52, ade2, arg8::hisG, lys2, leu2-3,112, mss51Δ::LEU2, PET309::3xHA nuc1Δ::KanMX4 [ρ^+]*[a]	(Zamudio-Ochoa et al., 2014)
FTC25	*Matα, ura3Δ, ade2 PET309-3xHA nuc1Δ::KanMX4 [ρ^+]*[a]	(Zamudio-Ochoa et al., 2014)
FTC26	*Matα, ura3-52, leu2-3, 112 lys2 arg8::hisG pet309Δ::LEU2 nuc1Δ::KanMX4 ρ^+, [ΔΣai]*[b]	(Zamudio-Ochoa et al., 2014)
YC133	*Matα, ura3Δ, ade2 nuc1Δ::KanMX4 [ρ^+]*[a]	(Zamudio-Ochoa et al., 2014)
SB7	*Matα, ura3Δ, ade2 MSS51-3xHA, [ρ^+]*[a]	(Perez-Martinez et al., 2003)
SB5	*Matα, ura3Δ, ade2 PET309-3xHA, [ρ^+]*[a]	(Zamudio-Ochoa et al., 2014)
XPM232	*Matα, ura3-52, leu2-3, 112 lys2 arg8::hisG pet309Δ::LEU2 [ρ^+, ΔΣai]*[b]	(Zamudio-Ochoa et al., 2014)
JPM16	*Matα, ura3Δ, ade2, pet309Δ::URA3, MSS51-3xHA [ρ^+]*[a]	This work
JPM22	*Matα, ura3Δ, ade2, PET309-3xHA, mss51Δ::KanMX4 [ρ^+]*[a]	This work
AZO53	*Matα, ura3-52, leu2-3, 112 lys2 arg8::hisG pet309Δ::LEU2, [ρ^+, cox1Δ::ARG8^m]*[a]	This work
AZO86	*Matα, ura3Δ, ade2 MSS51-3xHA, [ρ^+, cox1Δ::ARG8^m]*[a]	This work
YC140	*Matα, ura3Δ, ade2, pet309::URA3, MSS51-3XHA, [ρ^+, cox1Δ::ARG8^m]*[a]	This work
YC135	*Matα, ura3-52, leu2-3 112, lys2 arg8::hisG, pet309Δ::LEU2, mss51Δ::KANMX4 [ρ^+, cox1Δ::ARG8^m]*[a]	This work

[a] Mitochondrial genome is shown in brackets
[b] Mitochondrial *COX1* gene has no introns
All strains are isogenic or congenic to D273-10b

Table S2. Oligonucleotides Used in RT-PCR Experiments

Oligonucleotide	Sequence (5' → 3')	Reference
CO1E4R2[a]	TTGTAGCTCCAATTATTAATGGTAATAAATAG	(Zamudio-Ochoa et al., 2014)
VAR1R3[a]	TTATAATTATAAATTTGTAGATTTC	(Zamudio-Ochoa et al., 2014)
CO15´-400F[b]	CATATATATATATTTAATGATATTAATACTCTC	(Zamudio-Ochoa et al., 2014)
CO1R5-5'[b]	TAATCATCTTTGTACCATTTTTAC	(Zamudio-Ochoa et al., 2014)
VAR1F2[b]	AAATAATATTATAATAAGGATGC	This work
VAR1R2[b]	GTTGTAATTTATTATTAATATTCC	This work

[a] Oligonucleotide used for cDNA synthesis
[b] Oligonucleotide to amplify cDNA
