# Supplementary material for: Insights into the translational activation mechanisms of the COX1 mRNA in yeast mitochondria
Source: J Cell Sci. 2025 Sep 2;138(16):jcs263694. doi: 10.1242/jcs.263694 (PMC12450462; doi:10.1242/jcs.263694)
Supplement: Supplementary file 1 [file joces-138-263694_review_history.pdf]

Journal of Cell Science

Insights into the translational activation mechanisms of the *COX1* mRNA in yeast mitochondria

Angelica Zamudio-Ochoa, Yolanda Camacho-Villasana, Ulrik Pedroza-Dávila, Aldo E. Garcıa-Guerrero, Xochitl and Pérez-Martınez

DOI: 10.1242/jcs.263694

Editor: Ana Garcıa-Saéz
Review timeline
Original submission: 1 November 2024
Editorial decision: 6 December 2024
First revision received: 18 June 2025
Accepted: 14 July 2025

Original submission

First decision letter

MS ID#: jcs.263694

MS TITLE: Insights into the Translational Activation Mechanisms of the COX1 mRNA in Yeast Mitochondria

AUTHORS: Angelica Zamudio-Ochoa; Yolanda Camacho-Villasana; Aldo Eduardo Garcia-Guerrero; Xochitl Perez-Martinez

ARTICLE TYPE: Research Article

Dear Dr Perez-Martinez,

We have now reached a decision on the above manuscript.

To see the reviewers' reports and a copy of this decision letter, please go to:

As you will see, the reviewers raise a number of substantial criticisms that prevent me from accepting the paper at this stage. They suggest, however, that a revised version might prove acceptable, if you can address their concerns. If you think that you can deal satisfactorily with the criticisms on revision, I would be pleased to see a revised manuscript. We would then return it to the reviewers.

Reviewer 1

Advance summary and potential significance to field

In the work by Zamudio-Ochoa and coauthors, a great progress have been made in the field of mitochondrial translation regulation. In yeast, such regulation mainly occurs via so-called translational activators. Each of these proteins, alone or together with others, is able to selectively promote translation of a given mitochondrial mRNA. There are about 20 activators known, some of them interact with target mRNAs, some with mitochondrial ribosomes. However, molecular mechanisms of an activation itself remain unclear. In the present work, the authors show the association of two activators of COX1 mRNA, Mss51 and Pet309, to mitochondrial ribosomes. This

© 2025. Published by The Company of Biologists under the terms of the Creative Commons Attribution License (https://creativecommons.org/licenses/by/4.0/).

association seems not to depend on active COX1 mRNA translation. Based on the original findings, as well as on the previously obtained data, Zamudio-Ochoa and coauthors propose a mechanism of COX1 mRNA activation in yeast mitochondria.
In fact, this is a very first work containing a mechanistic insight on the processes of mitochondrial translation activation. The proposed model itself is not fully based on the experimental data but at least it has been proposed and can be verified further. Thus, on my opinion, this work is of high significance to the field. However, I see some defects in the experimental disign as well as some minor shortcomings. This is why I would recommend to revise the paper so that it becomes suitable for publication.

Comments for the author

Major point:
The main method you use to assess the activators assosiation to the mitochondrial ribosome is the ultracentrifugation in the discontinuous sucrose gradient. The gradient is then divided into seven fractions and analyzed by western blot. As the indicators of mitoribosomes presence, you used one protein of the small subunit and another one of the large subunit. Then, when you see the band of a target protein in the same fraction where both indicators are presented, you conclude that a target protein is assosiated with mitoribosomes.
From the results you present, it is difficult to conclude whether the target proteins are assosiated to the assembled ribosomes or to just small or large subunits. If you divide the material into just 7 fractions, then each of this fractions is too "wide" and may contain both assembled ribosomes and one of the separated subunits. In other words, if you see your protein in the fraction containing both indicator proteins, this could mean that your protein is assosiated to (1) assembled ribosomes or (2) just one of its subunits. At the same time, the question whether or not Mss51 and Pet309 are assosiated with the assembled ribosomes is of high importance since assembled ribosomes means translating ribosomes, and this finding, if robustly supported by experimental data, could greatly improve the significance of the proposed model. In this model, both Mss51 and Pet309 remain bound to mitoribosomes during the whole translation process which means that they must assosiate to the assembled ribosomes.
On my opinion, this approach, if used in more comprehensive manner, might give much more information that you have obtained. I would recommend to change the experimental procedure by increasing the number of fractions to 15-20. With this experimental setup, you could easily distinguish between (1) small subunits, (2) large subunits and (3) assembled ribosomes. To me, the easiest way to detect them all is to perform northern blots with the oligos complementary to the corresponding ribosomal RNAs. However, western blots with antibodies against the indicator proteins might also be enough.

Minor points:
1. Page 6, lines 26-28. The phrase "This construct was cloned into a centromeric plasmid (CEN) and expressed it episomally in a pet309Î" strain" looks strange, please rewrite.
2. Page 6, line 30. Please add references to Figures S1A and S2A after the statement that no respiratory defect in the strain bearing the PET309-3xHA construct was observed.
3. I would suggest to directly mention in the main text that the strains with pet309Î"nt and pet309Î"ct do not grow on glycerol which means that their mitochondria do not respire. Currently, these results are presented in the supplementary material only, and no reference to the corresponding panels of Figs S1 and S2 is given in the main text.
4. Page 10, line 41. The phrase part "...are critical for binding the COX1 mRNA and to associate with the mitoribosome..." looks strange, please rewrite.
5. Page 12, line 28, and page 14, line 47. In these two cases, you call Pet309 a ribosomal subunit which is defeinitely incorrect. If I understand right, you mean that it may be regarded as the constitutive mitoribosomal protein. Please rewrite.

<u>Reviewer 2</u>

Advance summary and potential significance to field

This paper by Zamudio-Ochoa et al. describes the nature of the interaction of the translation activators Pet309 and Mss51 with the mitochondrial ribosome. The paper clearly and unambiguously

© 2025. Published by The Company of Biologists under the terms of the Creative Commons Attribution License (https://creativecommons.org/licenses/by/4.0/).

shows that these proteins interact with the ribosome independently. Furthermore, their interaction with the mitoribosome is independent of mitoribosome binding to COX1 mRNA. The authors proposed a refined model of COX1 mRNA translation activation by the studied translation activators based on the data obtained. The work has been carried out very carefully and clearly, the data obtained are clearly presented and well described. The conclusions and assumptions are unquestionable. Overall, the article is well written, the experimental design is excellent, and the results are very interesting. The article can be published after revisions.

Comments for the author

Nevertheless, there are some minor shortcomings and suggestions for improvement of the paper. First, the small number of sucrose gradients fractions is surprising. Perhaps dividing the gradients into more fractions would have increased the resolution of the experiments. In addition, it might have been possible to answer the question of which subunit (SSU or LSU) Pet309 and Mss51 interact with, or whether they migrate in gradients only with associated, i.e. translating, mitoribosomes.
In the experiments on mRNA analysis in immunoprecipitates (Figure 4), the supernatant fraction (S) is mentioned in the figure caption but not directly shown in the images. Either correct photographs should be provided or, if these data are not important, the description of Figure 4 should be corrected.
The sedimentation experiment of Mss51 from strain rho0 (Figure 6B) does not look very convincing, as a weak signal is observed when stained with antibodies against the HA tag in the middle of the sucrose gradient. Perhaps more experiments should be performed with Mss51 overexpression in such a strain (similar to Pet309 overexpression in rho0 strain, Figure 1C).
In my opinion, Pet309 should not be referred to as a ribosomal subunit, as this term is generally accepted to apply to SSU and LSU. Perhaps the term "mitoribosomal protein" should be used.

<u>Reviewer 3</u>

Advance summary and potential significance to field

The authors investigate the basis of Pet309 and Mss51 as translational activators of COX1 mRNA, which forms the catalytic core of cytochrome c oxidase, in mitochondrial protein synthesis of the budding yeast. This study builds upon previous research from the group on the function of Pet309 and Mss51. In the current study, the authors investigate the mechanisms by which these two proteins interact with mitochondrial ribosomes to coordinate the synthesis of COX1. What is missing, however, is a mechanistic and spatial understanding on where these proteins interact with yeast mitochondrial ribosome. Considering a structure is solved for the yeast mitochondrial ribosome, this task is not too high of a requirement and would provide a greater understanding of Pet309 and Mss51 regulation of mitochondrial protein synthesis and how specificity is achieved.

Major points
1. The authors use sucrose gradients to investigate the association of Pet309-3XHA and Mss51-3xHA with mitochondrial ribosomes. A key control is missing to rule out co-migration from association with the mitoribosome. The clarified lysates should be treated with a nuclease before loading on to the gradient. The overexpression experiments with a 2 ï¤- plasmid in a rho-zero background are not sufficient.

2. Is the interaction of Pet309 with the mitochondrial ribosome dependent on translation? Treating the isolated mitochondria with a short pulse of puromycin would terminate nascent chain synthesis, so the association of Pet309 could be tested.

3. The authors report that the N-terminal region of Pet309 is required for interaction with the mitochondrial ribosome and COX1 mRNA binding. Has it been demonstrated that this mutant construct still localises to mitochondria?

4. A cross-linking experiment would be important to establish the location of Pet309 and Mss51 interaction on the mitochondrial ribosome. This spatial information is critical to establish the working models.

© 2025. Published by The Company of Biologists under the terms of the Creative Commons Attribution License (https://creativecommons.org/licenses/by/4.0/).

5. Pet309 does not appear to be conserved in Animals. However, in mammalian mitochondria there is a PPR protein (PTCD3) that stably associates with the mitochondrial ribosome. Since the authors have modeled Pet309 in Alpha fold is there any structural homology with the PTCD3 and in the process revealing a conserved function? This would be illuminating for understanding the evolution of mitochondrial protein synthesis as there are no mitochondrial translational activators in Metazoa.

6. Many elements of the proposed model COX1 translation are difficult to envision and far too speculative on how specificity is achieved. For example, in (3), how would Pet309 remain bound to the COX1 mRNA during translation elongation? A PPR protein continuously bound to the COX1 mRNA would impede translation elongation by creating a clash at the mRNA entry channel.
Minor points
1. Introduction, first paragraph, lines 19-21, this reviewer would disagree with the statement that the budding yeast is the most used system for mitochondrial translation. Over the last 15 years the number of research groups investigating human and mammalian mitochondrial protein synthesis has ballooned, especially with the relevance to human disease, and the number of published studies and mechanistic insight developed in those systems reflects this fact. Please delete the sentence as it is misleading to the average reader.

2. Introduction, first paragraph, l 27-28, the synchrony of translation regulation between mitochondrial and cytoplasmic ribosomes is only seen in budding yeast. The same group (Churchman lab) that published the yeast study (Couvillion et al. 2016) showed that this is not seen in mammalian cultured cells (Soto et al. 2022). Therefore, please amend the statement.

3. The potential relevance of Mss51 in human disease is not needed for the current study and unnecessary. Please remove the relevant text in the introduction and discussion.

4. The average reader of the journal will not be familiar with the MIOREX complex, so some elaboration on what it represents is in warranted.

5. The statistical analysis is not described properly nor is it apparent that assumptions of parametric statistics have been followed adequately. Performing parametric statistical analysis on percentages is not correct and is distorted because of the data distribution. Percentages need to be transformed before testing.

First revision

Author response to reviewers' comments

Reviewer 1: SUMMARY OF THE ADVANCE MADE IN THIS PAPER AND ITS POTENTIAL SIGNIFICANCE TO THE FIELD

In the work by Zamudio-Ochoa and coauthors, a great progress have been made in the field of mitochondrial translation regulation. In yeast, such regulation mainly occurs via so-called translational activators. Each of these proteins, alone or together with others, is able to selectively promote translation of a given mitochondrial mRNA. There are about 20 activators known, some of them interact with target mRNAs, some with mitochondrial ribosomes. However, molecular mechanisms of an activation itself remain unclear. In the present work, the authors show the association of two activators of COX1 mRNA, Mss51 and Pet309, to mitochondrial ribosomes. This association seems not to depend on active COX1 mRNA translation. Based on the original findings, as well as on the previously obtained data, Zamudio-Ochoa and coauthors propose a mechanism of COX1 mRNA activation in yeast mitochondria.
In fact, this is a very first work containing a mechanistic insight on the processes of mitochondrial translation activation. The proposed model itself is not fully based on the experimental data but at least it has been proposed and can be verified further. Thus, on my opinion, this work is of high significance to the field. However, I see some defects in the experimental design as well as some

© 2025. Published by The Company of Biologists under the terms of the Creative Commons Attribution License (https://creativecommons.org/licenses/by/4.0/).

minor shortcomings. This is why I would recommend to revise the paper so that it becomes suitable for publication.

SUGGESTIONS TO AUTHORS
Major point:
The main method you use to assess the activators assosiation to the mitochondrial ribosome is the ultracentrifugation in the discontinuous sucrose gradient. The gradient is then divided into seven fractions and analyzed by western blot. As the indicators of mitoribosomes presence, you used one protein of the small subunit and another one of the large subunit. Then, when you see the band of a target protein in the same fraction where both indicators are presented, you conclude that a target protein is assosiated with mitoribosomes.
From the results you present, it is difficult to conclude whether the target proteins are associated to the assembled ribosomes or to just small or large subunits. If you divide the material into just 7 fractions, then each of this fractions is too "wide" and may contain both assembled ribosomes and one of the separated subunits. In other words, if you see your protein in the fraction containing both indicator proteins, this could mean that your protein is assosiated to (1) assembled ribosomes or (2) just one of its subunits. At the same time, the question whether or not Mss51 and Pet309 are assosiated with the assembled ribosomes is of high importance since assembled ribosomes means translating ribosomes, and this finding, if robustly supported by experimental data, could greatly improve the significance of the proposed model. In this model, both Mss51 and Pet309 remain bound to mitoribosomes during the whole translation process which means that they must assosiate to the assembled ribosomes.
On my opinion, this approach, if used in more comprehensive manner, might give much more information that you have obtained. I would recommend to change the experimental procedure by increasing the number of fractions to 15-20. With this experimental setup, you could easily distinguish between (1) small subunits, (2) large subunits and (3) assembled ribosomes. To me, the easiest way to detect them all is to perform northern blots with the oligos complementary to the corresponding ribosomal RNAs. However, western blots with antibodies against the indicator proteins might also be enough.

> The experimental conditions we used are designed to analyze assembled mitoribosomes, as we do not use EDTA in our solutions (Aufschnaiter at al (2023) Methods. Mol. Biol.). We reinforced this fact in the results section, page 5 line 19-20 with the phrase: "*The conditions used in all our experiments preserved the assembled mitoribosome. No EDTA was added to dissociate the subunits*". Moreover, to demonstrate that in our experimental conditions a similar migration pattern of assembled ribosomes is equally observed in 7 or 14 fractions we performed additional gradient ultracentrifugation experiments collecting 14 fractions, as usually reported in other labs, where 12-15 fractions are commonly analyzed (Aufschnaiter at al (2023) Methods. Mol. Biol.; Choi & Barrientos (2021) Methods. Mol. Biol., Anderson et al (2022) Mol. Biol. Cell; Jeffri-Noelle Mays et al (2019) Nuc. Acids Res.; Hillman GA et al (2019) J. Biol. Chem, among others). This experiment is mentioned in the results section page 5 line 22-28 " *Since the integrity of the ribosomes was preserved, we decided to analyze seven fractions from the sucrose gradients instead of the 12 to 15 fractions that are commonly used, which are especially useful when analyzing disassembled subunits. The ribosome migration patterns were similar after analyzing both seven and fourteen fractions (Figure S3).*"

Minor points:

1. Page 6, lines 26-28. The phrase "This construct was cloned into a centromeric plasmid (CEN) and expressed it episomally in a pet309Δ strain" looks strange, please rewrite.

> We rewrote the sentence to *“This construct was cloned into a centromeric plasmid (CEN). Yeast pet309Δ cells were transformed with this plasmid for expression”* for better clarity. Page 5 Line 13-14.

2. Page 6, line 30. Please add references to Figures S1A and S2A after the statement that no respiratory defect in the strain bearing the PET309-3xHA construct was observed.

> We added the references in Page 5 Line 16.

© 2025. Published by The Company of Biologists under the terms of the Creative Commons Attribution License (https://creativecommons.org/licenses/by/4.0/).

3. I would suggest to directly mention in the main text that the strains with pet309Δnt and pet309Δct do not grow on glycerol which means that their mitochondria do not respire. Currently, these results are presented in the supplementary material only, and no reference to the corresponding panels of Figs S1 and S2 is given in the main text.

> We added the reference of each figure, Page 8 Line 15-16 for pet309Dct, and Page 8 Line 25 for pet309Dnt.

4. Page 9, line 31. The phrase part "...are critical for binding the COX1 mRNA and to associate with the mitoribosome..." looks strange, please rewrite.

> We changed the sentence to *“Overall, this data indicates that the first six PPR motifs of Pet309 are crucial for binding to the COX1 mRNA and interacting with the mitoribosome",* Page 9 Line 30.

5. Page 12, line 28, and page 14, line 47. In these two cases, you call Pet309 a ribosomal subunit which is definitely incorrect. If I understand right, you mean that it may be regarded as the constitutive mitoribosomal protein. Please rewrite.

> We agree, Pet309 is not a ribosomal protein, so we changed this statement to *“Pet309 constitutively associates with the mitoribosome”*. Page 14 Line 4.

Reviewer 2: SUMMARY OF THE ADVANCE MADE IN THIS PAPER AND ITS POTENTIAL SIGNIFICANCE TO THE FIELD
This paper by Zamudio-Ochoa et al. describes the nature of the interaction of the translation activators Pet309 and Mss51 with the mitochondrial ribosome. The paper clearly and unambiguously shows that these proteins interact with the ribosome independently. Furthermore, their interaction with the mitoribosome is independent of mitoribosome binding to COX1 mRNA. The authors proposed a refined model of COX1 mRNA translation activation by the studied translation activators based on the data obtained. The work has been carried out very carefully and clearly, the data obtained are clearly presented and well described. The conclusions and assumptions are unquestionable. Overall, the article is well written, the experimental design is excellent, and the results are very interesting. The article can be published after revisions.

SUGGESTIONS TO AUTHORS
Nevertheless, there are some minor shortcomings and suggestions for improvement of the paper. First, the small number of sucrose gradients fractions is surprising. Perhaps dividing the gradients into more fractions would have increased the resolution of the experiments. In addition, it might have been possible to answer the question of which subunit (SSU or LSU) Pet309 and Mss51 interact with, or whether they migrate in gradients only with associated, i.e. translating, mitoribosomes.

> In our experimental conditions only assembled mitoribosomes are present (Aufschnaiter at al (2023) Methods. Mol. Biol.). For simplicity we used 7 fractions instead of the usual 12-15 fractions. We reinforced this fact in the results section, page 5 line 19-20 with the phrase: " *The conditions used in all our experiments preserved the assembled mitoribosome. No EDTA was added to dissociate the subunits* ". Moreover, to demonstrate that in our experimental conditions a similar migration pattern of assembled ribosomes is equally observed in 7 or 14 fractions we performed additional gradient ultracentrifugation experiments collecting 14 fractions, as usually reported in other labs (Aufschnaiter at al (2023) Methods. Mol. Biol.; Choi & Barrientos (2021) Methods. Mol. Biol., Anderson et al (2022) Mol. Biol. Cell; Jeffri-Noelle Mays et al (2019) Nuc. Acids Res.; Hillman GA et al (2019) J. Biol. Chem). This is included as additional Figure S3. We also clarified this point by adding the phrase: " Since the integrity of the ribosomes was preserved, we decided to analyze seven fractions from the sucrose gradients instead of the 12 to 15 fractions that are commonly used, which are especially useful when analyzing disassembled subunits (Aufschnaiter at al, 2023; Choi & Barrientos, 2021; Anderson et al, 2022; Jeffri-Noelle Mays et al, 2019; Hillman GA et al, 2019). The ribosome migration patterns were similar after analyzing both seven and fourteen fractions (Figure S3)" to Results section, page 5, line 23.

In the experiments on mRNA analysis in immunoprecipitates (Figure 4), the supernatant fraction (S) is mentioned in the figure caption but not directly shown in the images. Either correct photographs

© 2025. Published by The Company of Biologists under the terms of the Creative Commons Attribution License (https://creativecommons.org/licenses/by/4.0/).

should be provided or, if these data are not important, the description of Figure 4 should be corrected.

> We corrected this observation in Figure 4, Page 31 Line 5-7.

The sedimentation experiment of Mss51 from strain rho0 (Figure 6B) does not look very convincing, as a weak signal is observed when stained with antibodies against the HA tag in the middle of the sucrose gradient. Perhaps more experiments should be performed with Mss51 overexpression in such a strain (similar to Pet309 overexpression in rho0 strain, Figure 1C).

> The observed stain in this figure corresponds to a real scratch on the original film and not a western blot signal. We added a comment noting this in the figure legend.

In my opinion, Pet309 should not be referred to as a ribosomal subunit, as this term is generally accepted to apply to SSU and LSU. Perhaps the term "mitoribosomal protein" should be used.

>We agreed that Pet309 is not a classical ribosomal protein, we simply stated Pet309 as a constitutively interactor of the mitoribosome. Page 14 Line 4.

Reviewer 3: Summary
The authors investigate the basis of Pet309 and Mss51 as translational activators of COX1 mRNA, which forms the catalytic core of cytochrome c oxidase, in mitochondrial protein synthesis of the budding yeast. This study builds upon previous research from the group on the function of Pet309 and Mss51. In the current study, the authors investigate the mechanisms by which these two proteins interact with mitochondrial ribosomes to coordinate the synthesis of COX1. What is missing, however, is a mechanistic and spatial understanding on where these proteins interact with yeast mitochondrial ribosome. Considering a structure is solved for the yeast mitochondrial ribosome, this task is not too high of a requirement and would provide a greater understanding of Pet309 and Mss51 regulation of mitochondrial protein synthesis and how specificity is achieved.

Major points
1. The authors use sucrose gradients to investigate the association of Pet309-3XHA and Mss51-3xHA with mitochondrial ribosomes. A key control is missing to rule out co-migration from association with the mitoribosome. The clarified lysates should be treated with a nuclease before loading on to the gradient. The overexpression experiments with a 2 μ plasmid in a rho-zero background are not sufficient.

> The association of Pet309 and Mss51 with the mitoribosome has been previously demionstrated (Salvatori et al (2020) Mol. Cell; Bridgers et al (2025) Biorxiv; Mays e tal (2019) Nucleic Ac. Res.; Da Silva et al (2017), Nucleic. Acids. Res; Verma et al (2021) MBC) and it is beyond the scope of this article to demonstrate it again. Nonetheless, we included a control by analyzing the migration of Pet309-3xHA and Mss51-3xHA in cells without mtDNA. We consider this sufficient evidence because in a rho 0 strain, there are no mitoribosomes, since Var1, an essential component of the small ribosomal subunit, is encoded by this mtDNA, together with the small and large rRNAs. If the migration of Pet309 and Mss51 were circumstantial and not a true association with the mitoribosome, then in the strain without mitoribosomes (rho0), the migration pattern of Pet309 and Mss51 should be the same as that in the WT strain. We explained this better by addition of the phrase: *"Since the large and small rRNAs, as well as the ribosomal protein Var1 are encoded in this genome, a ρ^o strain has no mitoribosomes."* in the results section, Page 6 Line 4-5.

2. Is the interaction of Pet309 with the mitochondrial ribosome dependent on translation? Treating the isolated mitochondria with a short pulse of puromycin would terminate nascent chain synthesis, so the association of Pet309 could be tested.

> Puromycin treatment releases the *COX1* mRNA from the ribosome. We demonstrated that interaction of Pet309 with the mitoribosome does not require the presence of the *COX1* mRNA (by using a delta-cox1 strain), and thereof, Pet309 does not require an active translation to bind to the ribosome.

© 2025. Published by The Company of Biologists under the terms of the Creative Commons Attribution License (https://creativecommons.org/licenses/by/4.0/).

3. The authors report that the N-terminal region of Pet309 is required for interaction with the mitochondrial ribosome and COX1 mRNA binding. Has it been demonstrated that this mutant construct still localises to mitochondria?

> Presence of Pet309-delta N in mitochondria was demonstrated in Figure S2. All experiments in this figure were performed using purified mitochondria by Nicodenz-gradients and ultracentrifugation. Indeed, this protein behaves as an inner membrane peripheral protein, as the WT Pet309 does. To avoid confusions, we wrote *"Purified mitochondria…"* in Figure legends S1E and S1F for *pet309Dct* strain, and Figure S2D-F for *pet309Dnt* strain.

4. A cross-linking experiment would be important to establish the location of Pet309 and Mss51 interaction on the mitochondrial ribosome. This spatial information is critical to establish the working models.

> Identifying exactly where Pet309 and Mss51 bind to the ribosome would be very interesting. Unfortunately, so far, attempts to identify the binding site of Pet309 and Mss51 on the ribosome, both by our group (failed experiments to address the reviewer's comments) and by others (Desai et al (2017) Science; Bridgers et al (2025), Biorxiv) have not been fruitful. We do not know the origin of these experimental difficulties. It might be that the interaction of these translational activators is with ribosomal proteins and/or fragments of rRNA, or some other reason. Unfortunately, our multiple attempts have not yielded any conclusive information. The inability to precisely determine the location of Pet309 and Mss51 on the ribosome does not interfere with our main conclusions: 1) Binding of Pet309 and Mss51 to the ribosome is independent of each other's presence. 2) The binding of these translational activators to the ribosome does not depend on the ribosome actively translating the *COX1* mRNA. 3) All three domains of Pet309 are required for the protein to be active, particularly, the N-terminal domain is required for both, binding to the *COX1* mRNA and to the ribosome. The other domains are inactive, although they can bind to the *COX1* mRNA. Knowing which proteins (and/or rRNA sequences) of the mitoribosome interact with Pet309 and Mss51 will be of great interest for future work.

5. Pet309 does not appear to be conserved in Animals. However, in mammalian mitochondria there is a PPR protein (PTCD3) that stably associates with the mitochondrial ribosome. Since the authors have modeled Pet309 in Alpha fold is there any structural homology with the PTCD3 and in the process revealing a conserved function? This would be illuminating for understanding the evolution of mitochondrial protein synthesis as there are no mitochondrial translational activators in Metazoa.

> The experimental structure of some PPR proteins in plants like PPR10 and THA8, and some PPR-containing proteins like PRORP1 and MORP9/RIP9 have been previously solved, showing the same super-helical pattern that AlphaFold predicts for Pet309 and other predicted PPR proteins. Due to the intrinsic RNA sequence-specificity that PPR proteins show, most studied PPR proteins are involved in RNA translational control, stability and editing. Therefore, PPR proteins share a role in RNA metabolism in all studied eukaryotes (For more reference see: Rovira AG, Smith AG (2019). Physiol Plant. However, a specific conserved function among PPR proteins involved in translation is hard to elucidate. PTCD3, MrpS27 and LRPPRC are PPR proteins related to mitochondrial translation in human (Davies et al (2009) FEBS Lett.; Davies et al (2012) FEBS Lett; Singh et al (2024) Nat. Struct. Mol. Biol.). However, none of these proteins have a specific role on the *COX1* mRNA, in contrast to what we observe for Pet309. We have tried in the past to compensate the absence of Pet309 by expression of the human LRPPRC without success, probably because each PPR protein has a specific mechanism of action, as specific are the protein and RNA interactors.

6. Many elements of the proposed model COX1 translation are difficult to envision and far too speculative on how specificity is achieved. For example, in (3), how would Pet309 remain bound to the COX1 mRNA during translation elongation? A PPR protein continuously bound to the COX1 mRNA would impede translation elongation by creating a clash at the mRNA entry channel.

> We agree with the reviewer in that interaction of Pet309 with the *COX1* mRNA throughout elongation would be difficult to envision and it is highly speculative. For clarity of our model, we decided to eliminate that proposal.

© 2025. Published by The Company of Biologists under the terms of the Creative Commons Attribution License (https://creativecommons.org/licenses/by/4.0/).

Minor points
1. Introduction, first paragraph, lines 19-21, this reviewer would disagree with the statement that the budding yeast is the most used system for mitochondrial translation. Over the last 15 years the number of research groups investigating human and mammalian mitochondrial protein synthesis has ballooned, especially with the relevance to human disease, and the number of published studies and mechanistic insight developed in those systems reflects this fact. Please delete the sentence as it is misleading to the average reader.

>We agreed with this comment, and we deleted the sentence to avoid misleading affirmations.

2. Introduction, first paragraph, l 27-28, the synchrony of translation regulation between mitochondrial and cytoplasmic ribosomes is only seen in budding yeast. The same group (Churchman lab) that published the yeast study (Couvillion et al. 2016) showed that this is not seen in mammalian cultured cells (Soto et al. 2022). Therefore, please amend the statement.

> We agree, a difference in translation synchronization exist between yeast and mammals. In the first paragraph, we described the genes encoded in mitochondria from *Saccharomyces cerevisiae*, and we stated that in this model translation is tightly regulated and synchronized with the assembly of nuclear-encoded subunits. We added the phrase *"This phenomenon does not seem to exist in mammalian mitochondria (Soto et al, 2022)"* Page 3 Line 13-14.

3. The potential relevance of Mss51 in human disease is not needed for the current study and unnecessary. Please remove the relevant text in the introduction and discussion.

> We agree that this information distracts from the main purpose of this study, so we deleted that text.

4. The average reader of the journal will not be familiar with the MIOREX complex, so some elaboration on what it represents is in warranted.

> We included a sentence to briefly explain the performed analysis and the explanation of the MIOREX acronym. We added the phrase *"Pet309 was previously detected in a mass-spectrometry analysis from isolated mitoribosomes, revealing that Pet309 interacts with the mitochondrial ribosome, and is part of large assemblies termed mitochondrial organization of gene expression (MIOREX) complexes"* in Page 5, Line 7-9

5. The statistical analysis is not described properly nor is it apparent that assumptions of parametric statistics have been followed adequately. Performing parametric statistical analysis on percentages is not correct and is distorted because of the data distribution. Percentages need to be transformed before testing.

> We changed our statistical analysis by calculating the Log2-fold change of the *COX1/15S* ratio relative to the control, followed by a one-way ANOVA, with a Geisser-Greenhouse correction, and a Tuckey's comparison test. We added this information to the Materials and Methods section on the Northern blot description.

Second decision letter

MS ID#: jcs.263694R1

MS Title: Insights into the Translational Activation Mechanisms of the COX1 mRNA in Yeast Mitochondria

© 2025. Published by The Company of Biologists under the terms of the Creative Commons Attribution License (https://creativecommons.org/licenses/by/4.0/).

Authors: Angelica Zamudio-Ochoa; Yolanda Camacho-Villasana; Aldo Eduardo Garcia-Guerrero; Xochitl Perez-Martinez; Ulrik Pedroza-Davila

Article Type: Research Article

Dear Dr Perez-Martinez,

I am happy to tell you that your manuscript has been accepted for publication in Journal of Cell Science, pending standard publication integrity checks.

Reviewer 1

Advance summary and potential significance to field

All the issues I have mentioned in my comment to the original manuscript version have been properly addressed in the revised version. My opinion is that, in its present form, the manuscript is suitable for publication.

© 2025. Published by The Company of Biologists under the terms of the Creative Commons Attribution License (https://creativecommons.org/licenses/by/4.0/).
